# Taxonomy and distribution pattern of the African rain forest butterfly genus *Euphaedra* Hübner *sensu stricto* with the description of three new subspecies of *Euphaedra cyparissa* (Cramer) and one of *E. sarcoptera* (Butler) (Lepidoptera, Nymphalidae, Limenitidinae, Adoliadini)

**DOI:** 10.3897/zookeys.298.4894

**Published:** 2013-05-10

**Authors:** Tomasz W. Pyrcz, Haydon Warren-Gash, Jadwiga Lorenc-Brudecka, Dieuwko Knoop, Philippe Oremans, Szabolcs Sáfián

**Affiliations:** 1 Zoological Museum of the Jagiellonian University, Ingardena 6, 30-060 Kraków, Poland; 2 98 Overstrand Mansions, Prince of Wales Drive, London SW11 4EU; 3 35, rue des Jacinthes, 6110 Montigny - le - Tilleul, Belgique; 4 Institute of Silviculture and Forest Protection, University of West Hungary, Sopron, Hungary; 5 Rijsberkamperweg 7, 8392TP Boyl, The Netherlands

**Keywords:** Afrotropical region, colour patterns convergence, *Euphaedra cyparissa*, *Euphaedra sarcoptera*, male and female genitalia, rain forests, subspecies, taxonomy

## Abstract

Updated data on the distribution, ecology and taxonomy of *Euphaedra cyparissa* (Cramer) and *Euphaedra sarcoptera* (Butler) are presented. Three new subspecies of *Euphaedra cyparissa* and one of *Euphaedra sarcoptera* are described and their geographic distribution is presented. The monophyly of the genus *Euphaedra sensu* Hecq is assessed based on morphological, in particular male and female genitalia, and behavioural traits. Possible evolutionary reasons for the convergence of colour pattern between the sympatric subspecies of *Euphaedra cyparissa* and *Euphaedra sarcoptera* are discussed.

## Introduction

Recent years have witnessed a rising interest in advanced studies focusing on species-rich African brush-footed butterflies (Nymphalidae). Examples include ongoing studies, combining molecular, ecological and morphological data, on the genus *Cymothoe* Hübner (Van Velzen et al. 2007, 2009, 2013) and *Bicyclus* Kirby used as one model group for modern research on genetics and evolutionary ecology (Monteiro and Pierce 2001; Conceição et al. 2011). From this perspective, one of the most speciose African butterfly genera, *Euphaedra* Hübner with nearly 200 recognized species, remains unexplored. This is due to the taxonomic complexity, but most of all to the unstable species-level systematics involved. The latter is a result of the tradition of basing *Euphaedra* taxonomy on simple descriptions of wing colour patterns. It is striking that descriptions of new species of *Euphaedra* have not usually been accompanied by in-depth morphological analysis, nor by ecological (with the honourable exception of Amiet 2004) or biogeographic support, not to mention genetic data (Hecq 1984, 1997, 1999, 2012; Faravel 2002). The morphology of female genitalia of *Euphaedra* remained until recently nearly completely unexplored and was not used with any alpha-taxonomy or in assessing the phylogenetic relations within the genus (Pyrcz et al. 2011). One of the major problems when dealing with species-level *Euphaedra* taxonomy is the huge variation in individual colour pattern evident within some species, on occasion more important to the naked eye than intraspecific differences, compounded by apparently rather frequent intraspecific hybridization producing viable individuals. This, coupled with wide geographic ranges, apparent pattern convergence between sympatric species and the lack of strong, quantitative characters, often impedes reliable recognition of species, which has led to the description of numerous species which are in fact merely individual variations of polymorphic species, or possibly natural hybrids (Hecq 2012). The above mentioned systematic problems effectively hampered any comprehensive studies within this group according to modern evolutionary biology approach, for example using molecular data (Wahlberg et al., in prep.). At this stage, the genus requires more detailed studies at the lower taxonomic level, such as species groups or subgenera, to help prepare the ground for a more broad based revision.

The subgenus *Euphaedra* was delimited by Hecq (1976) in an attempt to break down the extremely diverse genus *Euphaedra sensu* Hübner into several related, possibly monophyletic groups designated as subgenera. His work relied strongly on colour patterns, such as forewing subapical bands and hindwing underside elements as valid characters, and some characters of male genitalia, especially the configuration of cornuti on the vesica and shape of the apical part of valva. According to Hecq (1976) the subgenus *Euphaedra* is identified by the elongated forewing apex and the absence of red scaling on the hindwing underside. Subsequent authors, to a different degree, recognized the entities raised by Hecq, among others, D’Abrera (2004), Larsen (2005) and Vande Weghe (2010). The two species making up the subgenus *Euphaedra sensu* Hecq, *Euphaedra cyparissa* (Cramer 1775) and *Euphaedra sarcoptera* (Butler 1871), present some apparent morphological and, indeed, behavioural characters that set them apart from other congeners. Schultze (1920) pointed out their less developed, slender thorax. A striking colour pattern element - the pinkish ventral basal patch - present on the forewing in *Euphaedra sarcoptera* and absent in *Euphaedra cyparissa* makes the immediate separation between the two species unambiguous (Larsen 2005). They are sympatric through most of their range in West and Central Africa. However, *Euphaedra sarcoptera* occurs as far east as western Tanzania, whereas *Euphaedra cyparissa* does not extend into the Congo basin. Both species are widely polytypic. Distribution of the three subspecies of *Euphaedra cyparissa* described so far is puzzling, being either widely disjunct and/or without any apparent geographical pattern. Larsen (2005) discusses three or possibly four subspecies of *Euphaedra cyparissa* but in the headings of the species he highlights only two, the nominate and ssp. *tai* Hecq. In the accompanying text, however, he also mentions *aurata* Carpenter and suggests that the Central African Republic population possibly represents a separate subspecies. Vande Weghe (2010) simply illustrates all green specimen, presumably from Gabon, and basically quotes Larsen (op. cit).

## Material and methods

Adults of *Euphaedra* were collected using entomological nets and fruit-baited traps. Type specimens deposited in major collections were examined. Male and female genitalia were dissected and compared. Standard dissection protocols were applied, consisting in soaking the abdomens in a warm 10% KOH solution for 35 min., cleaning out of scales and internal organs in distilled water, and staining genital organs with chlorazole black. Genital preparations were placed in glycerol microvials, and pinned under their respective specimens. Photographs of adults were made with an Olympus E-500 digital camera and microscopic structures were photographed under an Olympus stereomicroscope SZX9 equipped with a Nikon Digital sight Ds-Fi1 camera. Plates were composed with Adobe Photoshop 9. Abbreviations used: FW: forewing; HW: hindwing; D: dorsum; V: venter; TL: Type locality.

### Collections consulted

**ABRI** African Butterfly Research Institute, Nairobi, Kenya

**BMNH** The Natural History Museum (formerly British Museum of Natural History), London, UK

**DK** Dieuwko Knoop, Tel Aviv, Israel and Boyl, The Netherlands

**GF** Gilles Faravel, Pradons, France

**HWG** Haydon Warren-Gash, Pressac, France

**MIIZ** Muzeum i Instytut Zoologii Polskiej Akademii Nauk, Warszawa, Poland

**MRAC** Musée Royal de l’Afrique Centrale, Tervuren, Belgium

**MZUJ** Muzeum Zoologiczne Uniwersytetu Jagiellońskiego, Kraków, Poland

**RW** Robert Warren (currently in DK)

**SZS** Szabolcs Sáfián, Sopron, Hungary

**SMTD** Senckenberg Naturhistorische Sammlungen Dresden Museum für Tierkunde

**PhO** Philippe Oremans, Montigny-le-Tilleul, Belgique

**TL** Torben Larsen (data base)

**TWP** Tomasz Wilhelm Pyrcz (to be deposited in MZUJ)

## Results

### Taxonomic accounts

#### Euphaedra cyparissa cyparissa

(Cramer)

http://species-id.net/wiki/Euphaedra_cyparissa_cyparissa

**Figure 1.**
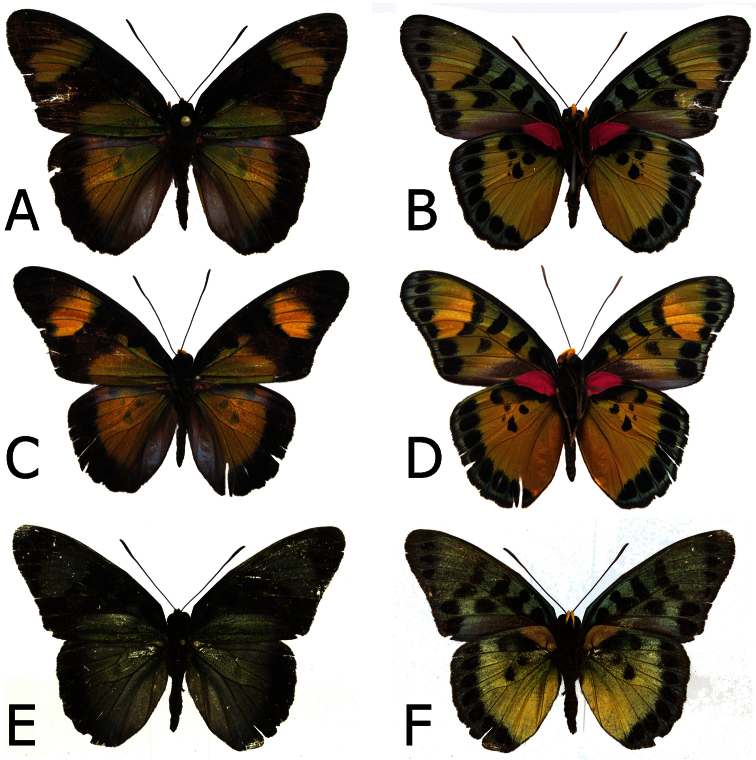
Adults, males: **A** *Euphaedra cyparissa cyparissa* Mount Swa, Liberia (dorsum) **B** *Euphaedra cyparissa cyparissa* Mount Swa, Liberia (venter) **C** *Euphaedra cyparissa nimbina* Mont Nimba, Guinea, holotype (dorsum) **D** *Euphaedra cyparissa nimbina* Mont Nimba, Guinea, holotype (venter) **E** *Euphaedra cyparissa tai* Tai, Ivory Coast (dorsum) **F** *Euphaedra cyparissa tai* Tai, Ivory Coast (venter).

Figs 1A, 1B , 3A, 3B , 9A

Papilio cyparissa Cramer, [1775], *in* Cramer, [1775–1776]: 63, pl. 39. figs D, E.
Papilio cato Fabricius, 1787: 12 (unnecessary replacement name)

##### Type-locality.

Sierra Leone

##### Material examined.

1 ♂: Sierra Leone, Guma, 01.03.1982, prep. genit. 10/09.05.2012, J. Lorenc; 1 ♀: same data, prep. genit. 11/09.05.2012, J. Lorenc; 1 ♀: Sierra Leone, Guma Valley, 400 m, 11.1991; 1 ♀: Sierra Leone, no locality, no date; 1 ♂: Ivory Coast, Abidjan (erroneous locality), 1966, S. Collins leg.; 1 ♀: same data (erroneous locality), ABRI; 6 ♂ and 2 ♀: Sierra Leone, Guma, 01.III.1982, H. Warren-Gash leg.; 1 ♂: Guinea, Diecke, 07.2000, HWG; 1 ♂ and 5 ♀: Sierra Leone, SMTD; 4 ♂: Guinée, Guinée forestière, Province de Yomou, Forêt classée de Diecke, 1-26.III.2003, leg. Ph. Leonard and E. Vingerhoedt; 3 ♂ and 1 ♀: same data but VI.2003; 2 ♂: same data but III.2005, PhO; 3 ♂ and 1 ♀: Guinée, Forêt classée de Diecké, GF; 2 ♂ and 2 ♀: Liberia, Mount Swa, Sz. Sáfián leg, MZUJ; 1 ♂: same data, ABRI.

##### Diagnosis.

Upperside colour black and apple green with a delicate yellow sheen, somewhat more prominent on the HWD median area.

##### Redescription.

**Male** (Figs 1A, 1B). Head: eyes lustrous, chocolate brown, naked; vertex black with a tuft of short, black hair; labial palpi covered with dense, short, bright yellow hairy scales; antennae half the length of costa, slender, gradually widening into a flattened club, in its widest part only twice as wide as shaft, dorsally black, ventrally bright yellow, covered with sparse sales along most of its length except for terminal segments. Thorax: black, dorsally sparsely covered with black and brown hair, denser laterally, with some violet blue scales on patagium and mesoscutum; tegulae covered with black, grey and brown hair; some longer and denser grey hair on metascutum; legs femora covered with black, tibiae and tarsi with sandy yellow scales. Abdomen: totally covered with dense, black scales, and some bluish scales on first, second and third segments dorsally (apparent only in fresh specimens), and over the entire length laterally. FWD: (length: 31–33 mm, mean: 32, n=9) with an elongated apical part, and gently convex outer margin; most of wing surface black; costa from base to apex dusted with dark blue; a rich green area from wing base to two-thirds of anal margin, marginally entering discal cell along lower part and at base of cell Cu1-Cu; a large, roughly oval rich green subapical patch with a marginal bluish sheen, extending widely from subcosta costa to vein M3, with sharply defined zigzagging basal limit, with an incision along vein M3, and a diffused outer limit; fringes grey. HWD oval with a gently scalloped outer margin; rich green, with a golden yellow overcast in outer one-third, a black marginal area gradually narrowing from roughly 4–5 mm at apex to 2 mm at tornus where heavily suffused with blue; fringes grey. FWV mostly bottle green, slightly lighter than on the FWD subapical patch, a series of large irregular black discal patches, at wing base, mid cell, cell end, an elongated black patch extending across cells M3-Cu1 and a large, roughly half-moon shaped patch in Cu1-Cu2; a diffused subapical golden yellow patch; a row of seven, roughly oval submarginal patches, two tornal patches in Cu1-Cu2 and Cu2-1/2A irregular and at least twice as big as the remainder. HWV golden green, with an elongated basal pinkish patch extending from costal margin to Rs to one-fourth costa, edged with black; four black median spots, two of which in discal cell, and a row of eight large, roughly oval black submarginal patches; marginal area darker bottle green and black. *Male genitalia* (Fig. 9A). Tegumen one and a half the length of uncus, considerably elongated basally; uncus slender, slightly arched downwards with a sharp tip; gnathos long, one-fourth longer than uncus; pedunculus prominent; saccus flattened in lateral view; valvae with a smooth dorsal surface; aedeagus the length of valva+saccus, tubular and straight, with a sharp distal extremity and prominent cornuti.

**Female** (Figs 3A, 3B): Sexual dimorphism slight, recognized from the male by the larger size (FW length: 40 mm, n=2). *Female genitalia*. Examined, but damaged and unsuitable for taxonomical use.

##### Range.

The known range of this subspecies extends from Sierra Leone, Liberia to SE Guinea. The pair in ABRI labeled as coming from Abidjan represent a conundrum. However, given the extensive research and collecting by one of the authors (HWG) in the Abidjan area without encountering any other specimens, we have concluded that they must be mislabeled. Larsen (2005) illustrated a male of the nominate subspecies identified as “green form from Ghana”, but this particular specimen comes from Sierra Leone (ABRI collection, examined). He also identified another specimen from Ghana as *Euphaedra cyparissa cyparissa*, which clearly does not belong to the nominate subspecies but to the new subspecies described below.

#### Euphaedra cyparissa tai

Hecq, 1986

http://species-id.net/wiki/Euphaedra_cyparissa_tai

Figs 1E, 1F , 4A, 4B , 9B , 10D

Euphaedra cyparissa tai [sic] Hecq, 1986: 42, figs. 11, 16, 17.

##### Type-locality.

Tai Forest (Forêt de Taï), Ivory Coast

##### Material examined.

12 ♂: Ivory Coast, Tai, 13–14.05.2000, S. Collins leg.; 1 ♂: same data, prep. genit. 07/09.05.2012, J. Lorenc; 1 ♀: same data; 1 ♀: same data, prep. genit. 08/09.05.2012, J. Lorenc; 1 ♂: same data but XII.1998, K. Adams leg., ABRI; 2 ♂: Ivory Coast, Tai, 12.I.1999, H. Warren-Gash leg.; 1 ♂: Ivory Coast, Tai, 13.I.1999, H. Warren-Gash leg.; 1 ♂ and 1 ♀: Ivory Coast, Tai, XII.1998, K. Adams; 2 ♂ and 1 ♀: Ivory Coast, Tai, 02.I.2000, H. Warren-Gash leg.; 1 ♂ and 1 ♀: Ivory Coast, Tai, 13.V.2000, H. Warren-Gash leg., HWG.

##### Diagnosis.

Upperside colour black and pine green.

##### Redescription.

**Male** (Figs 1E, 1F): Head, thorax and abdomen: identical to other subspecies. FWD: length 35–37 mm, mean: 36 mm, n=19 with an elongated apical part, and gently convex outer margin; most of wing surface black; costa from base to apex dusted with dark blue; a bottle green area from wing base to tornus, marginally entering discal cell; a large, roughly rectangular bottle green subapical patch with a bluish sheen, extending widely from subcostal to vein Cu1, with sharply defined zigzagging basal limit, without any incision along vein M3, and somewhat diffused outer limit; fringes grey. HWD oval with a gently scalloped outer margin; bottle green, gradually turning bluish green from vein M3 towards tornus and anal margin, with a black marginal area with a strong dark blue flush, gradually narrowing from roughly 4–5 mm at apex to 1 mm at tornus; fringes grey. FWV mostly bottle green, slightly lighter than on the upperside, a series of black spots, at wing base, mid cell, cell end, the latter two elongated, a half-moon one in mid cell Cu1-Cu2; two black elongated patches and four spots, two enclosing basally and distally a bottle green patch, and two large, roughly half-moon tornal patches in Cu1-Cu2 and Cu2-1/2A; HWV green with a strong yellow overcast gradually more prominent towards anal margin and tornus, with an elongated basal pinkish patch extending from costal margin to Rs to one-fourth costa, edged with black; two black discal spots, and a row of eight large, roughly oval black submarginal patches; marginal area darker bottle green and black. *Male genitalia* (Fig. 9B): Not differing noticeably from the nominotypical, except for a small, sharp apical tip on the valvae.

**Female** (Figs 4A, 4B): Sexual dimorphism slight, recognized from the male by the larger size (FW length: 43 mm, n=3). *Female genitalia* (Fig. 10D): Bursa copulatrix very large, roughly oval; no signa; ductus bursae (0.10 mm) approximately the same width over the whole length, half the length of bursa; antrum, here referred as to colliculum (Razowski 1996), slat like with gently folded edges, strongly sclerotized, one-third the length of ductus bursae, shorter than in other subspecies; ductus seminalis joins the colliculum at the entrance of ductus bursae; lamella postvaginalis slat-like; papillae anales three times as long (0.14 mm) as wide in lateral view, compressed in ventral view; apophyse posteriores as long as the width of papillae anales; von Siebold organ prominent (but smaller than in some species of the *Euphaedra ceres* group (Pyrcz et al. 2011).

##### Range.

The known distribution is limited to the Tai National Park in western Ivory Coast. The same pine green colour can be found in *Euphaedra sarcoptera styx* (see below) and also in the local population of *Euphaedra judith* Weymer, the latter probably deserving a separate subspecific status (Pyrcz et al. in prep.).

#### Euphaedra cyparissa nimbina

Pyrcz & Warren-Gash ssp. n.

http://species-id.net/wiki/Euphaedra_cyparissa_nimbina

Figs 1C, 1D , 3C, 3D , 9C , 10C

##### Type-locality.

Mount Nimba, Guinea

##### Material examined.

**Holotype** ♂: Guinea, Mont Nimba, III.1996, ABRI; Paratypes: (21 ♂ and 9 ♀): 1 ♂: Guinea, Mt. Nimba; 1 ♂: same data; 1 ♀: same data, prep. genit. 09/09.05.2012, J. Lorenc; 1 ♂: Ivory Coast, Mont Nimba, 01.1998, S. Collins leg., ABRI; 2 ♂: Guinée, Nimba, I.1998; 2 ♂ and 1 ♀: Guinée, Nimba, VI.1998; 1 ♀: Guinée, Nimba, X.1998; 1 ♂ and 1 ♀: Guinée, Labé, Fouta Djalon, X.1998; 1 ♂: Guinea, Sérédou, VI.1999; 5 ♂ and 1 ♀: Guinea, Mont Nimba, VI.2000, HWG; 1 ♂: Guinée, Mont Nimba, 02.V.1998, ex HWG; 1 ♂: same data but VI.2000; 1 ♀: same data; MZUJ; 7 ♂: Guinée, Mt. Nimba, VII.2004, E. Vingerhoedt leg.; 3 ♀: same data, PhO.

##### Diagnosis:

Upperside black and golden green, similar to *Euphaedra sarcoptera ferrea* ssp. n., little difference in shade between basal area and subapical patch, except for its slightly more prominent yellow shade.

##### Description.

**Male** (Figs 1C, 1D): Head, thorax and abdomen: identical to other subspecies. FWD: (length: 34–42 mm, mean: 38.5 mm, n=16) with an elongated apical part and gently convex outer margin; most of wing surface black; a golden green area from wing base to two-thirds of anal margin, covering lower one-third of discal cell, enclosing a mid discal cell black spot; a large, roughly rectangular golden yellow subapical patch with a greenish overcast along inner and outer margin, extending widely from subcosta to vein Cu1, with sharply defined zigzagging basal limit and somewhat diffused outer limit; fringes grey. HWD oval with a gently scalloped outer margin; uniform golden green, a black marginal area with a dark blue flush, roughly 4–5 mm wide with smooth and sharply defined basal edge (compared to more diffuse and zigzagging in the nominate); fringes grey. FWV mostly golden green, a series of black spots, at wing base, mid cell, cell end, the latter two elongated, a half-moon one in mid cell Cu1-Cu2; two black elongated patches and four spots, two enclosing basally and distally a rich yellow patch, and two large, roughly oval tornal patches in Cu1-Cu2 and Cu2-1/2A. HWV golden green with, with an elongated basal pinkish patch extending from costal margin to Rs to one-fourth costa, edged with black; two black discal spots, and a row of eight large, roughly oval black submarginal patches; marginal area darker bottle green and black. *Male genitalia* (Fig. 9C): Does not differ noticeably from the nominotypical, except for a small, sharp apical tip on the valvae.

**Female** (Figs 3C, 3D): Sexual dimorphism slight, recognized from the male by the larger size (43–50 mm, mean: 46 mm, n=5). *Female genitalia* (Fig. 10C): Bursa copulatrix large, oval; no signa; ductus bursae (0.19 mm) narrow, approximately the same width over the whole length, two-thirds the length of bursa; colliculum wide, slat like with folded edges, strongly sclerotized, one-third the length of ductus bursae; ductus seminalis joins the colliculum at the entrance of ductus bursae; lamella postvaginalis small, slat-like; papillae anales three times as long (0.14 mm) as wide in lateral view, compressed in ventral view; apophyse posteriores as long as the width of papillae anales; von Siebold organ prominent (but smaller than in some species of the *Euphaedra ceres* group (Pyrcz et al. 2011).

##### Etymology.

The subspecific epithet of this taxon derives from its type locality, the massif of Mount Nimba (1750 m) on the Ivory Coast – Guinea border.

##### Range.

The range is apparently disjunct with most specimens coming from the lower slopes of Mont Nimba and the surrounding area, and two collected in the Fouta Djalon range in northern Guinea, the latter record needs confirmation.

#### Euphaedra cyparissa aurantina

Pyrcz & Oremans ssp. n.

http://species-id.net/wiki/Euphaedra_cyparissa_aurantina

**Figure 2.**
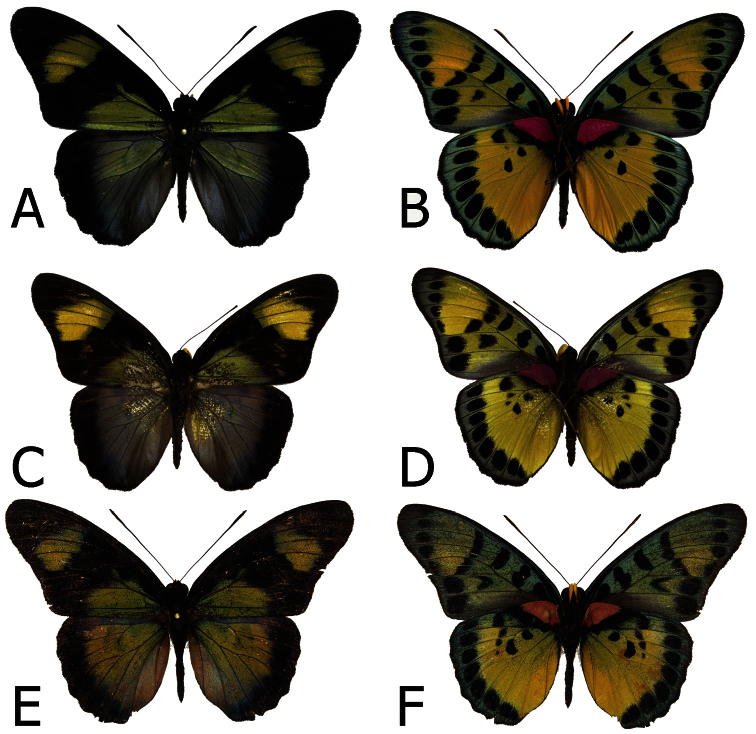
Adults, males: **A** *Euphaedra cyparissa aurantina* Bibiani, Ghana, holotype (dorsum) **B** *Euphaedra cyparissa aurantina* Bibiani, Ghana, paratype (venter) **C** *Euphaedra cyparissa aurata* Isheri, Nigeria (dorsum) **D** *Euphaedra cyparissa aurata* Isheri, Nigeria (venter) **E** *Euphaedra cyparissa nominalina* Mongoumba, R. C. A., holotype (dorsum) **F** *Euphaedra cyparissa nominalina* Mongoumba, R. C. A., holotype (venter).

**Figure 3.**
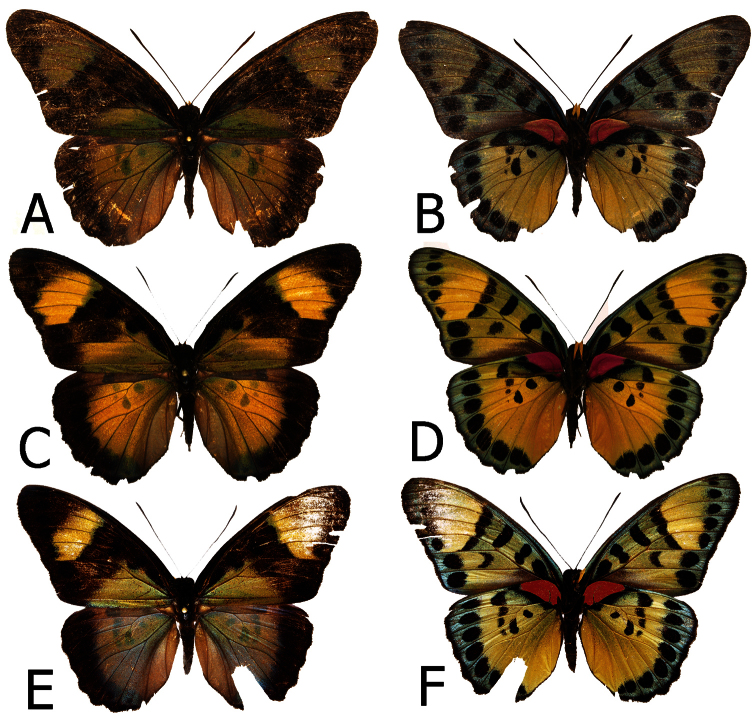
Adults, females: **A** *Euphaedra cyparissa cyparissa* Guma, Sierra Leone (dorsum) **B** *Euphaedra cyparissa cyparissa* Guma, Sierra Leone (venter) **C** *Euphaedra cyparissa nimbina* Mont Nimba, Guinea, paratype (dorsum) **D** *Euphaedra cyparissa nimbina* Mont Nimba,Guinea, paratype (venter) **E** *Euphaedra cyparissa aurantina* Atewa Forest, paratype (dorsum) **F** *Euphaedra cyparissa aurantina* Atewa Forest, paratype (venter).

Figs 2A, 2B , 3E, 3F , 9D , 10A, 10B

##### Type-locality.

N. Awasso, Bibiani, Western Region, Ghana

##### Material examined.

**Holotype** ♂: Ghana, Western, Bibiani, N. Awasso, 250–400 m, 02.IV.2007, MZUJ; Paratypes (127 ♂ and 29 ♀): 7 ♂: Ivory Coast, Abengourou, XI.1993, S. Collins leg., (1 ♂: prep. genit. 03/09.05.2012, J. Lorenc); 1 ♂: Ivory Coast, Abengourou, 13.V.1973; 1 ♀: Ivory Coast, Abengourou, 04.XII.1970, J. Kielland leg., prep. genit. 03/09.05.2012, J. Lorenc; 1 ♀: Ivory Coast, Abengourou; 1 ♀: Ivory Coast, Abengourou, S. Collins leg.; 1 ♂: Ivory Coast, Danane, IV.1973, S. Collins leg.; 1 ♀: same data but IV.1977; 1 ♀: same data but IV.1970 (all 3 specimens from « Danane » possibly mislabeled); 3 ♂: Ghana, Ankasa, XI.2003, Vorgas/Yevu leg.; 1 ♀: Ghana, Bia, XII.2003, Vorgas/Yevu leg.; 1 ♀: same data, prep. genit. 06/09.05.2012, J. Lorenc; 13 ♂: Ghana, Bia, XII.2003, Vorgas/Yevu leg.; 7 ♂: Ghana, Bia, III.2003; 1 ♀: Ghana, Bibiani, IV.2007, Vorgas/Yevu leg.; 1 ♂: Ghana, Bibiani, IV.2007, SY leg.; 1 ♀: Ghana, Bonkro, XI.2005, Vorgas/Yevu leg.; 5 ♂: Ghana, Cape 3 Points, I.2004, R. Vorgas leg.; 1 ♀: same data but I.2006, 1 ♂: Ghana, Kibi, XII.1996, S. Collins leg.; 1 ♂: Ghana, Kibi, S. Collins leg.; 1 ♂: Ghana, Mate, II.2002, Vorgas/Yevu leg.; 1 ♂: same data but III.2002; 1 ♂: Ghana, Mpasaso, VII.2004, Vorgas/Yevu leg.; 1 ♀: same data but V.2004, prep. genit. 02/09.05.2012, J. Lorenc; 1 ♂: Ghana, Nkwakwa, 04.IX.2000; 1 ♀: Ghana, Nkwakwa, IV.2000, S. Collins leg.; 3 ♂: Ghana, Sagamase, Kibi, X.2010, S. Collins leg.; 3 ♂: same data but XII.2000; 2 ♂: same data but IX.2003; 2 ♂: same data but I.2002; 1 ♂: same data but I.2001; 3 ♂: same data but XII.1997; 1 ♂: same data but IV.2003; 1 ♂: same data but VII.1998; 1 ♀: same data but I.2002; 1 ♀: same data but I.2003; 11 ♂: Ghana, Tano Ofin, III.2002, Vorgas/Yevu leg.; 1 ♂: same data, prep. genit. 01/09.05.2012, J. Lorenc; 13 ♂: same data but I.2004; 3 ♂: same data but II.2002; 1 ♂: same data but X.2009; 1 ♂: same data but IX.2002; 1 ♂: same data but XI.2003; 1 ♂: same data but IX.2001; 1 ♂: same data but III.2001; 4 ♂: same data but II.2001, R. Vorgas leg.; 1 ♂: same data but Vorgas/Yevu leg.; 1 ♂: same data but S. Collins leg.; 1 ♂: same data but XII.2001, S. Collins leg.; 4 ♂: same data but III.2001; 1 ♂: Ghana, Tano Ofin, I.2011; 1 ♀: same data but IV.2011; 2 ♀: same data but III.2002, Vorgas/Yevu leg.; 1 ♀: same data but II.2002; 1 ♀: same data but II.2001, R. Vorgas leg.; all ex coll. ABRI; 2 ♂: Ghana, Kyebi (Kibi) District, Eastern Region, Segyimaase, forestry access road, Atewa Range, 29.III-04.IV.2005, Sáfián, Sz., Csontos, G. and Kormos, B. leg.; 1 ♂: same data but 13–21.X.2007, Sáfián, Sz. leg., SZS; 1 ♀: Ghana, Eastern Region, Asuom Amanfrom, Amanfrom Forest, Kade District, 20–24.III.2005, Sáfián, Sz., Csontos, G. and Kormos, B. leg., SZS; 1 ♂: Ghana, Western, Bibiani, N. Awasso, 250–400 m, 01.IV.2007; 1 ♀: Ghana, Eastern Region, Atewa Forest, Sagymasse trail, 400–500 m, 16–27.X.2008, G. Csontos and Sz. Sáfián leg., MZUJ; 1 ♀: Ivory Coast, Banco, 11.XII.1999, H. Warren-Gash leg.; 1 ♀: Ivory Coast, South-West, Ft de Dassioko, 31.XII.1997, H. Warren-Gash leg.; 1 ♂: Ivory Coast, Foret d’Azagny, 10.III.2001, H. Warren-Gash leg.; 1 ♂: Ivory Coast, Foret d’Azagny, 06.I.2001, H. Warren-Gash leg., HWG; 14 ♂ and 7 ♀: Kibi, Ghana, XII.2012, local collector leg., Ph.O; 2 ♂ and 1 ♀: same data, MZUJ.

##### Diagnosis.

Upperside black and golden green with a yellow subapical patch strongly suffused with green, compared to the slightly suffused patch in *Euphaedra cyparissa aurata*.

##### Description.

**Male** (Figs 2A, 2B): Head, thorax and abdomen: identical to other subspecies. FWD: (length: 34–38 mm, mean: 37 mm, n=4): with an elongated apical part, and gently convex outer margin; most of wing surface black; costa from base to apex dusted with dark blue; a yellow green area with a golden sheen from wing base to two-thirds of anal margin, slightly entering discal cell and base of cell Cu1-Cu2; a large, roughly rectangular yellow subapical patch heavily dusted with green scales, with sharply defined zigzagging basal limit and somewhat diffused outer limit; fringes grey. HWD oval with a gently scalloped outer margin; predominantly golden green, gradually turning bluish green from vein M3 towards tornus and anal margin, with a blackish marginal area with a strong dark blue flush, gradually narrowing from roughly 4–5 mm at apex to 1 mm at tornus, leaving a dark blue submarginal dot in cell Cu1-Cu2; fringes grey. FWV mostly green yellow except for a large rich yellow subapical patch with some greenish overcast in apical part, a series of black spots, at wing base, mid cell, cell end, the latter two elongated, a half-moon one in mid cell Cu1-Cu2; two black elongated patches and four spots, two enclosing basally and distally a mostly rich yellow patch suffused with green yellow in its apical part, and two large, roughly square tornal patches in Cu1-Cu2 and Cu2-1/2A; HWV mostly rich yellow with an elongated basal pinkish patch extending from costal margin to Rs to one-third costa, edged with black; two black discal spots, and a series of smaller patches forming a gradually shrinking row from mod costa to discal cell end, a row of eight large, mostly rectangular black submarginal patches; marginal area steely blue. *Male genitalia* (Fig. 9D): Not differing noticeably from the nominotypical subspecies.

**Female** (Figs 3E, 3F): Sexual dimorphism slight, recognized from the male by the larger size (FW length: 43 mm, n=3). *Female genitalia* (Figs 10A, 10B): Bursa copulatrix large, oval; no signa; ductus bursae (0.21–0.24 mm) longer than in other subspecies, narrow, approximately the same width over the whole length, same length as bursa; colliculum slat like with gently folded edges, strongly sclerotized, one-third the length of ductus bursae; ductus seminalis joins the colliculum at the entrance of ductus bursae; lamella postvaginalis small, slat-like; papillae anales three times as long (0.9–0.13 mm) as wide in lateral view, compressed in ventral view; apophyse posteriores as long as the width of papillae anales; von Siebold organ prominent (but smaller than in some species of the *Euphaedra ceres* group (Pyrcz et al. 2011).

##### Etymology.

The epithet of this taxon is an allusion to its resemblance to the next subspecies, *Euphaedra cyparissa aurata*.

##### Range.

A widely distributed subspecies found in coastal forests in the Ivory Coast and Ghana, and also in inland forests in eastern Ivory Coast and Ghana.

#### Euphaedra cyparissa aurata

Carpenter, 1895

http://species-id.net/wiki/Euphaedra_cyparissa_aurata

**Figure 4.**
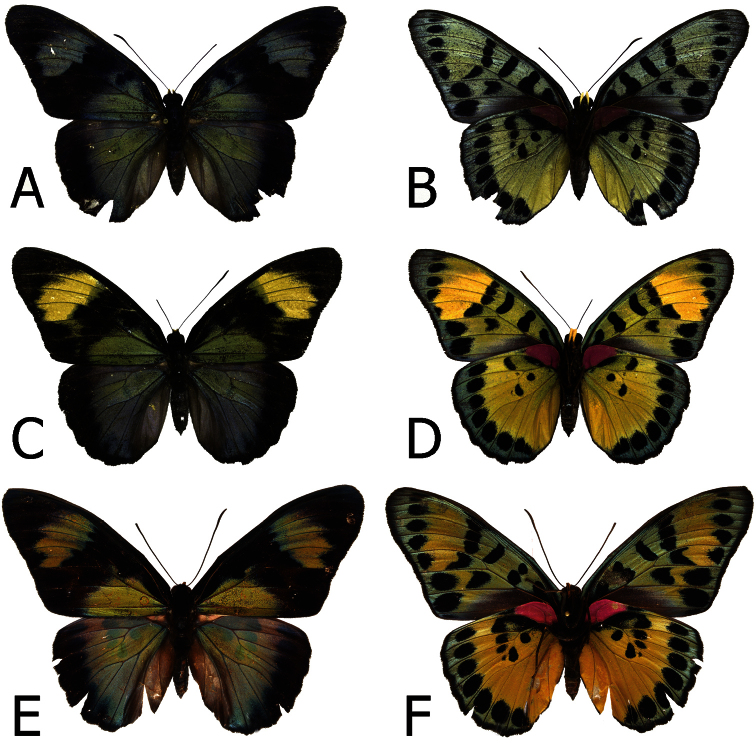
Adults, females: **A** *Euphaedra cyparissa tai* Tai, Ivory Coast (venter) **B** *Euphaedra cyparissa tai* Tai, Ivory Coast (venter) **C** *Euphaedra cyparissa aurata* Isheri, Nigeria (dorsum) **D** *Euphaedra cyparissa aurata* Isheri, Nigeria (venter) **E** *Euphaedra cyparissa nominalina* Borgen Dolphin Platform, paratype (dorsum) **F** *Euphaedra cyparissa nominalina* Borgen Dolphin Platform, paratype (venter).

Figs 2C, 2D , 4C, 4D , 9E , 10E

Euphaedra cyparissa var. *aurata* Carpenter, 1895: 305.
Euphaedra cyparissa aurata Hecq, 1999: 4, 5

##### Type-locality.

Lokoja, Nigeria

##### Material examined.

1 ♂: Nigeria, Butatong, Calabar, 14.XI.1995, S. Collins leg.; 1 ♀: same data but 14–18.XI.1995; 2 ♂: Nigeria, east, T. Davey leg.; 1 ♀: same data; 1 ♂: Nigeria, Lagos, IX.1957, J. Boorman leg.; 1 ♀: same data but X.1962; 3 ♂: Nigeria, Mamu Forest, IV.1966, S. Collins leg.; 1 ♀: same data; 8 ♂: Nigeria, Obudu, 09–12.XI.1995, S. Collins leg.; 1 ♂: same data, prep. genit. 12/09.05.2012, J. Lorenc; 2 ♀: same data; 1 ♀: same data, prep. genit. 13/09.05.2012, J. Lorenc; 1 ♂: Nigeria, Okrasa, Calabar, S. Collins leg.; 1 ♀: Nigeria, Old Ekuri Calabar, 24–28.XI.1995, S. Collins leg.; 2 ♀: Nigeria, Olokomeji, IX.1957, J. Boorman leg.; 2 ♂: Cameroon, Ebogo, IV.1983, S. Collins leg., prep. genit. 16/09.05.2012, J. Lorenc, ABRI; 3 ♀: Nigeria, Isheri F., XII.1989, II-III.1990, A. A. Knoop Lindeboom leg; 2 ♂: Nigeria, Oban XI.1995, T. B. Larsen leg.; 1 ♂: Nigeria, Anambra F. R., VII.1962, St. Leger leg.; 6 ♂: Nigeria, Isheri F., IV.1988, II.1989, IV.1989, XII.1989, II.1990, A. A. Knoop Lindeboom leg.; 1 ♂: Nigeria, Isheri F., II.1991, D. P. Knoop leg. DK; 2 ♂: Nigeria, Isheri Forest, 29.III.1989, A. Knoop leg.; 3 ♂: same data but 09.V.1990; 1 ♂: same data but 23.I.1990; 1 ♂: same data but 14.II.1990; 1 ♂: same data but 09.IV.1990; 2 ♀: same data but I.1990; 1 ♀: same data but 10.III.1990; 1 ♀: same data but 05.IV.1989; 1 ♀: same data but 18.II.1990; 1 ♂: Nigeria, Olokomeji, 16.V.1988, A. Knoop leg.; 1 ♂: same data but 20.IV.1988; 1 ♀: same data; 1♂: Nigeria, Anambra State, Nsukka, 28.IX.1982, J. Wojtusiak leg.; 1 ♂: same data but 01.II.1983; 2 ♂: no data, MZUJ; 1 ♂: Nigeria, Olokomeji, 13.V.1989, leg. A. Knoop; 1 ♀: same locality, 20.IV.1988, leg. A. Knoop, PhO; 1 ♀: Gabon, Mondah, VIII.1993, leg. G. Faravel, GF.

##### Diagnosis.

Upperside colour black and mint green with a golden yellow subapical patch, usually with little or no green overcast.

##### Redescription.

**Male** (Figs 2C, 2D): Head, thorax and abdomen: identical to other subspecies. FW length 29–41 mm, mean: 36 mm, n=42 (one exceptionally small specimen, 25 mm, was examined). FWD: with an elongated apical part, and gently convex outer margin; most of wing surface black; costa from base to apex dusted with dark blue; a yellow green area from wing base to two-thirds of anal margin, slightly entering discal cell and base of cell Cu1-Cu2; a large, roughly rectangular rich yellow subapical patch dusted along the edges with yellow green scales, with sharply defined zigzagging basal limit and somewhat diffused outer limit; fringes grey. HWD oval with a gently scalloped outer margin; predominantly steely bluish with some green yellow scaling in discal cell and in cells M1-M2 and M2-M3, with a blackish marginal area with a strong dark blue flush, gradually narrowing from roughly 4–5 mm at apex to 1 mm at tornus, leaving a dark blue submarginal dot in cell Cu1-Cu2; fringes grey. FWV mostly green yellow except for a large rich yellow subapical patch with some greenish overcast in apical part, a series of black spots, at wing base, mid cell, cell end, the latter two elongated, a half-moon one in mid cell Cu1-Cu2; two black elongated patches and four spots, two enclosing basally and distally a mostly rich yellow patch suffused with green yellow in its apical part, and two large, roughly square tornal patches in Cu1-Cu2 and Cu2-1/2A; HWV mostly rich yellow with an elongated basal pinkish patch extending from costal margin to Rs to one-third costa, edged with black; two black discal spots, and a series of smaller patches forming a gradually shrinking row from mod costa to discal cell end, a row of eight large, mostly rectangular black submarginal patches; marginal area steely blue. *Male genitalia* (Fig. 9E): Not differing noticeably from the nominotypical except for a small, sharp apical tip on the valvae.

**Female** (Figs 4C, 4D): FW length: 40–48 mm. Sexual dimorphism slight, recognized from the male by the larger size (FW length: 39–47mm, mean: 44mm, n=16). *Female genitalia* (Fig. 10E) Bursa copulatrix large, rounded; no signa; ductus bursae (0.16 mm) narrow, approximately the same width over the whole length, two-thirds the length of bursa; colliculum slat like with gently folded edges, strongly sclerotized, half the length of ductus bursae; ductus seminalis joins the colliculum at the entrance of ductus bursae; lamella postvaginalis small, slat-like; papillae anales three times as long (0.11 mm) as wide in lateral view, compressed in ventral view; apophyse posteriores as long as the width of papillae anales; von Siebold organ prominent (but smaller than in some species of the *Euphaedra ceres* group (Pyrcz et al. 2011).

##### Range.

Found in the rain forests throughout southern Nigeria, western Cameroon and Gabon. An individual (a female) collected by Faravel in Mondah, Gabon, is tentatively associated with this subspecies although it is lighter orange on the FW and HWD, and is therefore similar to *Euphaedra cyparissa nimbina* and also to *Euphaedra sarcoptera nipponicorum*, with which it was confused by Vande Weghe (2010). More sampling from southern Cameroon and Gabon is needed to evaluate the subspecific status of this population.

#### Euphaedra cyparissa nominalina

Pyrcz & Knoop ssp. n.

http://species-id.net/wiki/Euphaedra_cyparissa_nominalina

Figs 2E, 2F , 4E, 4F , 9F , 10F

Euphaedra cyparissa [Cramer], Vande Weghe, 2010: 115, fig. 15 (male, dorsum), 304–305.

##### Type-locality.

Mongoumba, Central African Republic

##### Material examined.

**Holotype** ♂: Central African Republic, Mongoumba, VII.1969, ABRI; Paratypes (5 ♂ and 4 ♀): 1 ♂: Central African Republic, Bangui, VIII.2000, S. Collins, prep. genit. 14/09.05.2012, J. Lorenc; 1 ♂: Central African Republic, near Bangui, 12.II.1969, J. Kielland leg.; 2 ♂: Central African Republic, Bomoloto, R. Loanye, VII.1985, S. Collins leg.; 1 ♀: Central African Republic, Mongoumba, 19.VII.1969, S. Collins leg.; 1 ♀: same data prep. genit. 15/09.05.2012, J. Lorenc; 1 ♀: Central African Republic, no locality, ABRI; 1 ♀: Off Shore Nigeria, Platform Borgen Dolphin, 4°18'98"35N, 8°22'00"32E, VIII. 2005, ex coll. P. Kowalski, MZUJ; 1 ♂: Cameroon, SE, Moloundou, 500 m, 25.VII.1989, E. Joly leg., PhO.

##### Diagnosis.

Upperside colour black and olive drab, same as in the nominate *Euphaedra cyparissa*, compared to golden green in *Euphaedra cyparissa aurata* and *Euphaedra cyparissa aurantina*, or pine green in *Euphaedra cyparissa tai*; differing from the widely separated geographically nominate *Euphaedra cyparissa* only in the slightly wider FWD subapical patch towards costal margin.

##### Description.

**Male** (Figs 2E, 2F): Head, thorax and abdomen: identical to other subspecies. FWD: (length 32–36 mm, mean: 34.9 mm, n=6) with an elongated apical part, and gently convex outer margin; most of wing surface black; costa from base to apex dusted with dark blue; a rich green area from wing base to three-fourths of anal margin, entering discal cell along lower part from base to root of vein Cu1, enclosing a black mid cell spot; a large, roughly rectangular bottle green subapical patch with a bluish sheen, extending widely along costa, with sharply defined zigzagging basal limit, without any notable incision along vein M3, and a diffused outer limit; fringes grey. HWD oval with a gently scalloped outer margin; rich green, gradually turning bluish green from vein M3 towards tornus and anal margin gradually narrowing from roughly 4–5 mm at apex to 2 mm at tornus; fringes grey. FWV mostly bottle green, slightly lighter than on the FWD subapical patch, a series of black spots, at wing base, mid cell, cell end, the latter two elongated, extending across cells M3-Cu1 and Cu1-Cu2; a row of seven, roughly oval submarginal patches, two tornal patches in Cu1-Cu2 and Cu2-1/2A twice as big as the remainder. HWV golden green, with an elongated basal pinkish patch extending from costal margin to Rs to one-fourth costa, edged with black; two black discal spots, and a row of eight large, roughly oval black submarginal patches; marginal area darker bottle green and black. *Male genitalia* (Fig. 9F): Not differing noticeably from the nominotypical.

**Female** (Figs 4E, 4F): Sexual dimorphism slight, recognized from the male by the larger size (FW length 50 mm, n=1). *Female genitalia* (Fig. 10F): Bursa copulatrix large, rounded; no signa; ductus bursae (0.22 mm) narrow, approximately the same width over the whole length, two-thirds the length of bursa; colliculum slat like with gently folded edges, strongly sclerotized, half the length of ductus bursae; ductus seminalis joins the colliculum at the entrance of ductus bursae; lamella postvaginalis small, slat-like; papillae anales three times as long (0.12 mm) as wide in lateral view, compressed in ventral view; apophyse posteriores slightly longer than the width of papillae anales; von Siebold organ prominent (but smaller than in some species of the *Euphaedra ceres* group (Pyrcz et al. 2011).

##### Etymology.

The epithet of this taxon is an allusion to its resemblance to the nominotypical subspecies.

##### Range.

On present knowledge, the range of this subspecies is limited to the CAR and eastern Cameroon, though its distribution pattern indicates it also occurs in adjacent areas of the DRC. The individual collected on an offshore platform in the Gulf of Guinea is an extraordinary finding. It is also a proof that *Euphaedra cyparissa* is prone to long distance dispersal. Other butterfly species collected on the same platform were typical dispersalists, including *Danaus*, *Acraea* and *Melanitis*. It is nonetheless surprising to find in that area an individual which clearly matches the phenotype of *Euphaedra cyparissa nominalina*,whereas the distribution pattern would rather suggest the presence of the subspecies *Euphaedra cyparissa aurata* along the Atlantic coast of Cameroon.

#### Euphaedra sarcoptera sarcoptera

(Butler, 1871)

http://species-id.net/wiki/Euphaedra_sarcoptera_sarcoptera

**Figure 5.**
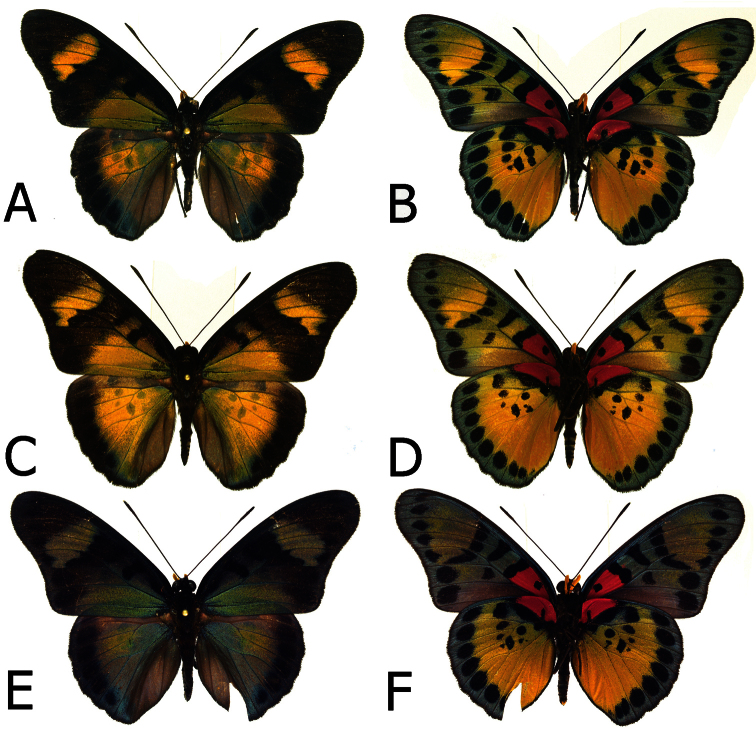
Adults, males: **A** *Euphaedra sarcoptera sarcoptera* male, Bibiani, Ghana (dorsum) **B** *Euphaedra sarcoptera sarcoptera*, Bibiani, Ghana (venter) **C** *Euphaedra sarcoptera ferrea* Mont Nimba, Guinea, holotype (dorsum) **D** *Euphaedra sarcoptera ferrea* Mont Nimba, Guinea, holotype (venter) **E** *Euphaedra sarcoptera styx*, Tai, Ivory Coast (dosum) **F** *Euphaedra sarcoptera styx*, Tai, Ivory Coast (venter).

Figs 5A, 5B , 7A, 7B , 11A

Romaleosoma sarcoptera Butler, 1871: 81, pl. 31, fig. 2.
Euphaedra sarcoptera (Butler); Aurivillius, 1925: 186.

##### Type-locality.

Ghana “Gold Coast”

##### Material examined.

1 ♂: Western Ghana, Bibiani, N. Awaso, 250–400 m, 09.IV.2007, ABRI; 1 ♂: same data, prep. genit. 04/20.06.2012, J. Lorenc; 1 ♀: Nsukka, Anambra State, Nigeria, 04.XII.1982, leg. J. Wojtusiak, prep. genit. 02/20.06.2012, J. Lorenc; 1 ♀: Banco N. P., Ivory Coast, 18.IX.1999, H. Warren-Gash leg., prep. genit. 05/20.06.2012, J. Lorenc, MZUJ; 1 ♂: Ghana, Oda Big Tree, XII.2010, Sáfián, Sz. leg., SZS; 1 ♂: Ghana, Eastern Region, Asuom Amanfrom, Amanfrom Forest, Kade District, 20–24.III.2005, Sáfián, Sz., Csontos, G. and Kormos, B. leg., SZS; 1 ♂: Ivory Coast, Alepe-Yaya, 06.II.1999, H. Warren-Gash leg.; 1 ♂: Ivory Coast, Alepe-Yaya, 12.XII.1999, H. Warren-Gash leg. 1 ♂: Ivory Coast, Lamto, 05.XI.2000, H. Warren-Gash leg.; 2 ♂ and 2 ♀: Ivory Coast, Alepe, 21.I.2000, H. Warren-Gash leg.; 1 ♀: Ivory Coast, Alepe, 07.I.2001, H. Warren-Gash leg., HWG; 1 ♂ and 1 ♀: Ivory Coast, Teapleu, V.1977; 10 ♂ and 3 ♀: Guinée, Guinée forestière, Province de Yomou, Forêt classée de Diecke, VI.2003, Eric Vingerhoedt leg.; 1 ♂: Cameroon, Douala, IX.1996, leg. P. Prouvost; 1 ♂: Cameroon, no exact locality, II.1997, E. Vingerhoedt, 1 ♂: Cameroon, Ebogo, I.1994, T. Garnier leg., PhO; 40 ♂ and 28 ♀: Ghana; 2 ♂ and 2 ♀: Ivory Coast; 1 ♂: S. Nigeria, Ewohimi, 29.XI.55, J. Boorman; 1 ♂: no label; 2 ♂: Cameroon, Ebogo, Mbalmayo, X.2001; 1 ♂: Cameroon, Ebogo, II.1994, S. Collins leg., ABRI; 1 ♂: Ghana, Kibi Atewa Mts., II.2007., J. Boersma; 1 male ♂: Nigeria, Ewohimi, I.1955, J. St. Leger leg., DK; 2 ♂: Nigeria, Sapoba, III.1967, J. Riley leg., ex Cornes and Riley coll., DK; 2 ♂ and 2 ♀: Liberia, Mount Swa, Sz. Sáfián leg, MZUJ; 1 ♂: same data, ABRI; 1 ♂: same data, SSS.

##### Diagnosis.

Upperside ground colour predominantly golden green, with the exception of the light yellow subapical patch.

##### Redescription.

**Male** (Figs 5A, 5B): Head: eyes lustrous, golden brown, naked; vertex black with a tuft of short, black hair with a bluish sheen; labial palps covered with dense, short, bright yellow hairy scales; antennae half the length of costa, slender, gradually widening into a flattened club, in its widest part only twice as wide as shaft, dorsally and ventrally all black, covered with sparse white and black sales along most of its length except for terminal segments. Thorax: black, dorsally sparsely covered with black and brown hair, denser laterally, with some violet blue scales on patagium and mesoscutum; tegulae covered with black hair; some longer and denser black hair on metascutum; legs femora, tibiae and tarsi with black scales. Abdomen: totally covered with dense, black scales, ventrally with some grey scales. Wings (FW length: 35–39 mm, mean: 37 mm, n=8) with an elongated apical part, and gently convex outer margin; most of wing surface black; costa from base to apex dusted with dark blue; a golden green area from wing base to two-thirds of anal margin, covering lower half of discal cell; a prominent mid discal cell black round spot; a large, elongated light yellow subapical patch suffused with green, extending widely from subcosta costa to vein Cu1, with a nearly straight basal limit marked by an incision along vein M3, and a diffused outer limit; fringes grey and black. HWD oval with a gently scalloped outer margin; rich golden green, with a diffuse golden postdiscal patch and a strong shiny blue overcast in outer one-third from vein Cu2 to anal margin; submarginal and marginal area black with a strong blue sheen gradually narrowing from roughly 4 mm at apex towards tornus and transforming into rectangular patches, the last of which in Cu2-1-2A detached; fringes grey and black. FWV golden green, slightly lighter than on the FWD; basal to mi-cell area purple; a series of black patches, a rounded postbasal spot, a wide mid cell bar, a similar bar along discal cell distal margin, a series of half-moon shaped patches along basal limit of a diffused subapical light yellow patch, a large half-moon shaped patch in mid cell Cu1-Cu2, and a series of submarginal patches, five smaller from costa to cell M3-Cu1 and two larger, oval, in Cu1-Cu2 and Cu2-1-2A. HWV golden yellow, with an elongated basal purple patch extending from costal margin to Rs to one-fourth costa, edged with black; six black median spots, four of which in discal cell, and a row of eight large, roughly oval black submarginal patches; marginal area darker bottle green and black. *Male genitalia* (Fig. 11A): Tegumen same length as uncus, slightly arched; uncus stout, tip slightly curved downwards; gnathos long, same length as uncus; pedunculus prominent; saccus flattened in lateral view; valvae with a smooth dorsal surface and blunt apex; aedeagus as long as valvae, tubular and straight, with a sharp distal extremity and prominent cornuti.

**Female** (Figs 7A, 7B): Sexual dimorphism slight except for the larger size of the female (FW length: 41–45 mm, mean: 43 mm, n=5) and duller dorsal colours. *Female genitalia*: not examined.

##### Range.

The nominate subspecies is widely distributed from central Liberia across Ivory Coast, Ghana and Nigeria and into Cameroon and northern Gabon, in both in perhumid coastal forests and drier forests in the interior. It is phenotypically quite stable throughout with the notable exception of the Mount Swa population in Liberia where all green individuals were detected flying alongside typically patterned ones. Such an individual variation may be explained by phenotype selection to a heterogenous environment of patchy rain forest and savanna. Nevertheless, it remains to be confirmed that the green and typical morphs are indeed syntopic and we cannot exclude the possibility that the green morph represents a separate parapatric subspecies.

#### Euphaedra sarcoptera styx

Larsen & Warren-Gash, 2003

http://species-id.net/wiki/Euphaedra_sarcoptera_styx

**Figure 6.**
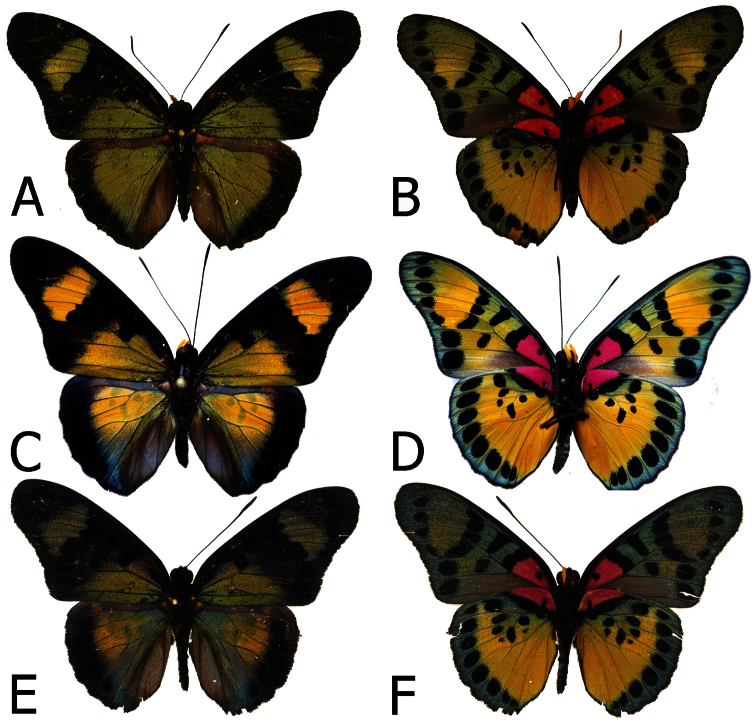
Adults, males: **A** *Euphaedra sarcoptera cyparissoides* holotype (dorsum) **B** *Euphaedra sarcoptera cyparissoides* holotype (venter) **C** *Euphaedra sarcoptera nipponicorum* Mbandaka, Congo (dorsum) **D** *Euphaedra sarcoptera nipponicorum* Mbandaka, Congo (venter) **E** *E. sarcoptera sarcoptera-styx* cline, Mount Swa, Liberia (dorsum) **F** *E. sarcoptera sarcoptera-styx* cline, Mount Swa, Liberia (venter).

**Figure 7.**
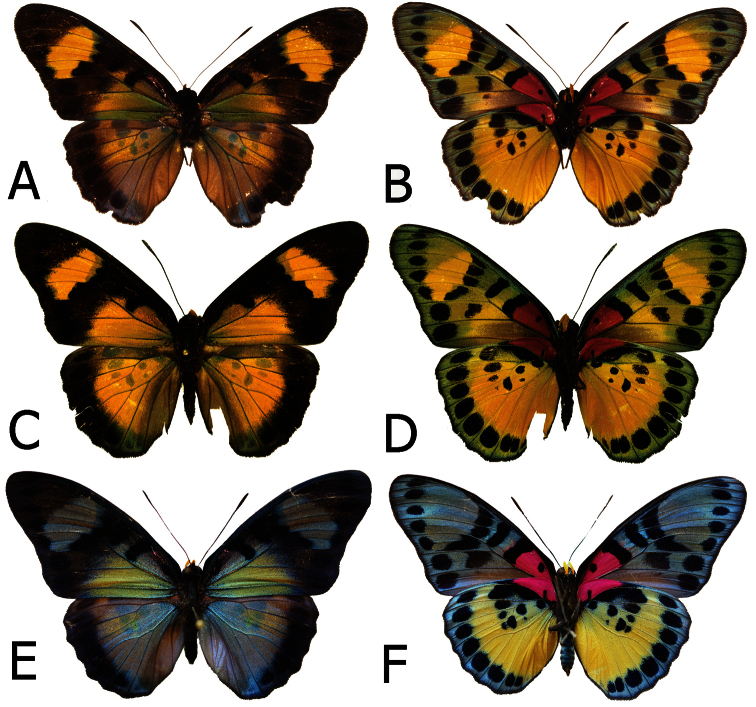
Adults, females: **A** *Euphaedra sarcoptera sarcoptera* Nsukka, Nigeria (dorsum) **B** *Euphaedra sarcoptera sarcoptera* Nsukka, Nigeria (venter) **C** *Euphaedra sarcoptera ferrea* Mont Nimba, Guinea, paratype (dorsum) **D** *Euphaedra sarcoptera ferrea* Mont Nimba, Guinea, paratype (venter) **E** *Euphaedra sarcoptera styx* Tai, Ivory Coast (dorsum) **F** *Euphaedra sarcoptera styx* Tai, Ivory Coast (venter).

Figs 5E, 5F , 6E, 6F , 7E, 7F , 11C

Euphaedra sarcoptera styx Larsen & Warren-Gash, 2003: 97.

##### Type-locality.

Tai Forest, Ivory Coast

##### Material examined.

1 ♂: Ivory Coast, Tai, 12.I.1999, H. Warren-Gash leg.; 3 ♂: Ivory Coast, Tai, 02.I.2000, H. Warren-Gash leg.; 1 ♂: Ivory Coast, Tai, XII.1998, K. Adams leg.; 1 ♀: Ivory Coast, Tai, 01–10.IV.2001, H. Warren-Gash leg., HWG; 1 ♂: Ivory Coast, Moyen Cavally, Tai Forest, IV.2001, prep. genit. 03/09.11.2012, J. Lorenc, HWG; 1 ♂: same data but 02.I.2000, MZUJ; 1 ♂: Tai, Ivory Coast, 13–14.VIII.2001, S. Collins; 1 ♂: same data but 13.IX.2001; 1 ♂: same data but 13–14.V.2000 (paratype *Euphaedra sarcoptera styx*); 1 ♂: Tai Forest, H. Warren-Gash leg., 12.1.1999 (paratype *Euphaedra sarcoptera styx*); 1 ♂: Tai Ivory Coast, 13–14.V.2000, S. Collins *leg*.; 1 ♀: same data but 13–14.VIII.2001, S. Collins leg., ABRI.

##### Diagnosis.

Upperside ground colour black and pine green.

##### Redescription.

**Male** (Figs 5E, 5F, 6E, 6F): Head, thorax and abdomen same to the nominate subspecies. Wings: FW (length 36–39 mm, mean: 37.8 mm, n=6) with an elongated apical part, and gently convex outer margin; most of wing surface black; costa from base to apex dusted with dark blue, suffusing a black mid-discal cell black patch; pine green area from wing base to tornus, covering most of Cu2-1A and half of Cu1-Cu2; a large, elongated lawn green subapical patch, extending widely from subcosta costa to vein Cu1, with the basal limit marked by an incision along vein M3, and a diffused outer limit, not reaching outer margin; fringes grey and black. HWD oval with a gently scalloped outer margin; pine green grey except for the row of submarginal black, roughly rectangular patches, with a strong blue sheen gradually narrowing from roughly 4 mm at apex to cell Cu1-Cu2; fringes grey and black. FWV pine green, with a delicate golden overcast in subapical area; basal to mid-cell area pink; a series of black patches, a rounded postbasal spot, a wide mid cell bar, a similar bar along discal cell distal margin, four rectangular and one elongated patches in postdiscal area, a half-moon shaped patch in basal area of cell Cu1-Cu2, and a series of submarginal patches, five smaller from costa to cell M3-Cu1 and two larger, roughly oval, in Cu1-Cu2 and Cu2-1-2A. HWV golden yellow dusted with pine green in median area, with an elongated basal pink patch extending from costal margin to Rs to one-third costa, edged with black; six or seven median spots, four of which in discal cell, the one along costal margin considerably larger than the remainder, and a row of eight large, roughly oval black submarginal patches; marginal area darker pine green and black. *Male genitalia* (Fig. 11C): Tegumen same length as uncus, with a flat dorsal surface; uncus stout, aligned with the dorsal surface of shoulder, tip sharp, both tegumen and uncus longer than in the nominate subspecies; gnathos long, same length as uncus; pedunculus prominent; saccus flattened in lateral view; valvae with a smooth dorsal surface and blunt apex; aedeagus as long as valvae tubular and straight, with a sharp distal extremity, slightly longer than in the nominate subspecies, and prominent cornuti.

**Female** (Figs 7E, 7F): Sexual dimorphism slight except for the larger size of the female (FW length: 40–41 mm, mean: 40.5 mm, n=2). *Female genitalia*: not examined.

##### Range.

Known distribution is limited to the Tai National Park in western Ivory Coast.

#### Euphaedra sarcoptera ferrea

Pyrcz & Warren-Gash ssp. n.

http://species-id.net/wiki/Euphaedra_sarcoptera_ferrea

Figs 5C, 5D , 7C, 7D , 11E , 12B

##### Type-locality.

Mount Nimba, Guinea

##### Material examined.

**Holotype** ♂: Guinée (Guinea), Mont Nimba, VI.1998, ex HWG, MZUJ; Paratypes (7 ♂and 4 ♀): 5 ♂and 1 ♀: Guinée, Mont Nimba, VI.1998; 1 ♀: same data but VI.2000, HWG; 2 ♂: same data but 02.V.1998, ex HWG; 1 ♀: same data but 02.V.1998, ex HWG, prep. genit. 01/09.11.2012, J. Lorenc, 1 ♀: Ivory Coast, Azagny N. P., 13.VIII.1999 (mislabeled), H. Warren-Gash leg., MZUJ.

##### Diagnosis.

Upperside ground colour golden yellow, same as in *Euphaedra sarcoptera nipponicorum*, differing from the golden green of the nominate subspecies, pine green of *Euphaedra sarcoptera styx* or apple green of *Euphaedra sarcoptera cyparissoides*, differing from *Euphaedra sarcoptera nipponicorum*, widely separated geographically,only in the narrower FWD subcapical elongated golden yellow patch.

##### Description.

**Male** (Figs 5C, 5D): Head, thorax and abdomen same as in the nominate subspecies. FW (length 38–40 mm, mean: 39 mm, n=6) with an elongated apical part, and gently convex outer margin; most of wing surface black, suffused with blue except for outer margin and apex, a black mid-discal cell black patch; golden green area from wing base to four-fifths of anal margin, covering most of discal cell; a large, elongated golden green subapical patch, extending from subcosta costa to vein Cu1, with the basal limit marked by an incision along vein M3, and a diffused outer edge, not reaching outer margin; fringes grey and black. HWD oval, compressed towards tornus, with a gently scalloped outer margin; golden green grey except for a black submarginal and marginal area, gradually narrowing from roughly 4 mm at apex towards tornus, where suffused with greenish blue scales; fringes grey and black. FWV pine green, lustrous, with a yellow overcast, except for the marginal area, more prominent in subapical area where forming a diffused elongated patch; basal to mid-cell area pink; a mid-cell bar, and a similar one along discal cell distal margin; four black, half-moon shaped patches along basally to the subapical yellow area, an elongated patch in mid cell Cu1-Cu2, and a series of submarginal patches, five smaller from costa to cell M3-Cu1 and two larger, oval, in Cu1-Cu2 and Cu2-1-2A. HWV golden yellow dusted with pine green along costa, with an elongated basal pink patch extending from costal margin to Rs to one-third costa, edged with black; six or seven median spots, four of which in discal cell, the one along costal margin considerably larger than the remainder; a row of eight large, roughly oval black submarginal patches; marginal area pine green and black. *Male genitalia* (Fig. 11E): Tegumen same length as uncus, with a flat dorsal surface; uncus stout, slightly arched, with a sharp tip pointing downwards, tegument and uncus approximately the same length as in the nominate subspecies; gnathos long, same length as uncus; pedunculus prominent; saccus flattened in lateral view; valvae with a smooth dorsal surface with an elongated distal extremity ending with a sharp tip, differing in this respect from the blunt valvae of the nominate subspecies and *styx*; aedeagus as long as valvae tubular and straight, with a sharp distal extremity, sensibly longer than in the nominate subspecies or *styx*, and prominent cornuti.

**Female** (Figs 7C, 7D): Head, thorax and abdomen abdomen same as in the nominotypical subspecies. Wings: FW (length 42-46 mm, n=3) with an elongated apical part, and gently convex outer margin; most of wing surface black; costa from base to apex dusted with dark blue; a golden yellow area from wing base to two-thirds of anal margin, covering lower half of discal cell; a prominent mid discal cell black round spot; a large, elongated golden yellow subapical patch, extending widely from subcosta costa to vein Cu1, with an irregular basal limit marked by an incision along vein M3, and a diffused outer limit, not reaching outer margin; fringes grey and black. HWD subtriangular with a gently scalloped outer margin; golden yellow, from Rs to costal margin dull brown; submarginal and marginal area black with a strong blue sheen, gradually narrowing from costa, where some 4 mm wide, to tornus, where blue takes over black; fringes grey and black. FWV green yellow, except for basal one-third where free of green and in subapical area where forming a large, diffuse patch; basal to median area purple; a series of black patches, a small rounded postbasal spot, a wide mid cell bar, a similar bar along discal cell distal margin, four patches along basal limit of a diffused subapical light yellow area, two outer triangular, two central rectangular, a large irregularly shaped patch in mid cell Cu1-Cu2, and a series of submarginal patches, five smaller from costa to cell M3-Cu1 and two larger, one rounded and one oval, in Cu1-Cu2 and Cu2-1-2A. HWV golden yellow, with an elongated basal purple patch extending from costal margin to Rs to one-fourth costa, edged with black; six black median spots, three of which in discal cell, and a row of eight large, roughly oval black submarginal patches; marginal area darker bottle green and black. *Female genitalia* (Fig. 12B): Bursa copulatrix very large, larger than the rest of genital body, oval, no signa; ductus bursae narrow, gradually widening towards the opening of bursa, one-third the length of bursa; colliculum narrow, slat like with folded edges,, one-third the length of ductus bursae; ductus seminalis joins the colliculum at the entrance of ductus bursae; lamella postvaginalis very small, slat-like; papillae anales three times as long as wide in lateral view, compressed in ventral view, apophyse posteriores one and half as long as the width of papillae anales; von Siebold organ prominent.

##### Etymology.

The subspecific epithet of this taxon derives from iron in Latin, *ferrum*. The type locality, the massif of Mount Nimba on the Ivory Coast – Guinea border, is rich in iron ore. Iron ore mining is a serious threat for the unique ecosystem of this mountainous area of West Africa.

##### Range.

Distribution limited to the Mount Nimba area. Considered that southern Ivory Coast was extensively sampled for several years, also by one of the authors (HWG), we believe that one paratype was labeled as from “Azagny” by error.

#### Euphaedra sarcoptera cyparissoides

Hecq, 1979

http://species-id.net/wiki/Euphaedra_sarcoptera_cyparissoides

Figs 6A, 6B

Euphaedra sarcoptera cyparissoides [sic] Hecq, 1979: 31.

##### Type-locality.

Ketta Forest, Ouesso, Moyen Congo

##### Material examined.

**Holotype** ♂: Ketta Forest (Ouesso), A.E.F. Moyen Congo, T. H. E. Jackon, VII.1959; 2 ♂: same data as the holotype, MRAC; 1 ♂: S Cameroon, Mintom, II.2009, P. A., ABRI.

##### Diagnosis.

Upperside ground colour black and apple green.

##### Redescription.

**Male** (Figs 6A, 6B): Head, thorax and abdomen same as in the nominate subspecies. Wings: FW (length 38–39 mm, n=2) with an elongated apical part, and gently convex outer margin; most of wing surface black almost completely outcasting a black mid-discal cell black patch; apple green area from wing base to three-fourth of anal margin, marginally entering the base of Cu2-1A and discal cell; a large, elongated apple green subapical patch a shade lighter, extending widely from subcosta costa to vein Cu1, with the basal limit marked by an incision along vein M3, and a diffused outer limit, not reaching outer margin; fringes grey and black. HWD oval, compressed towards tornus, with a gently scalloped outer margin; apple green grey except for the grey costal and black submarginal area gradually narrowing from roughly 4 mm at apex to cell Cu1-Cu2; fringes grey and black. FWV apple green, with a golden overcast in subapical area where forming a diffused patch; basal to mid-cell area pink; a series of black patches, a rounded postbasal spot, a wide mid cell bar, a similar bar along discal cell distal margin, four rectangular and one elongated patch in postdiscal area, a streak in basal area of cell Cu1-Cu2, and a series of submarginal patches, five smaller from costa to cell M3-Cu1 and two larger, roughly oval, in Cu1-Cu2 and Cu2-1-2A. HWV golden yellow dusted with apple green in median area, with an elongated basal pink patch extending from costal margin to Rs to one-third costa, edged with black; six or seven median spots, four of which in discal cell, the one along costal margin considerably larger than the remainder, and a row of eight large, roughly oval black submarginal patches; marginal area dark green and black. *Male genitalia*: not examined.

**Female**: Not examined

##### Range.

This subspecies was described from Ouesso in Congo, and apart from the holotype there are only a couple of other specimens known. Hecq (1979) mentions it from RCD and Cameroon. We could confirm its presence in SE Cameroon based on a specimen in ABRI. The reports from RDC might well refer to the next subspecies.

#### Euphaedra sarcoptera nipponicorum

(Carcasson), 1965

http://species-id.net/wiki/Euphaedra_sarcoptera_nipponicorum

**Figure 8.**
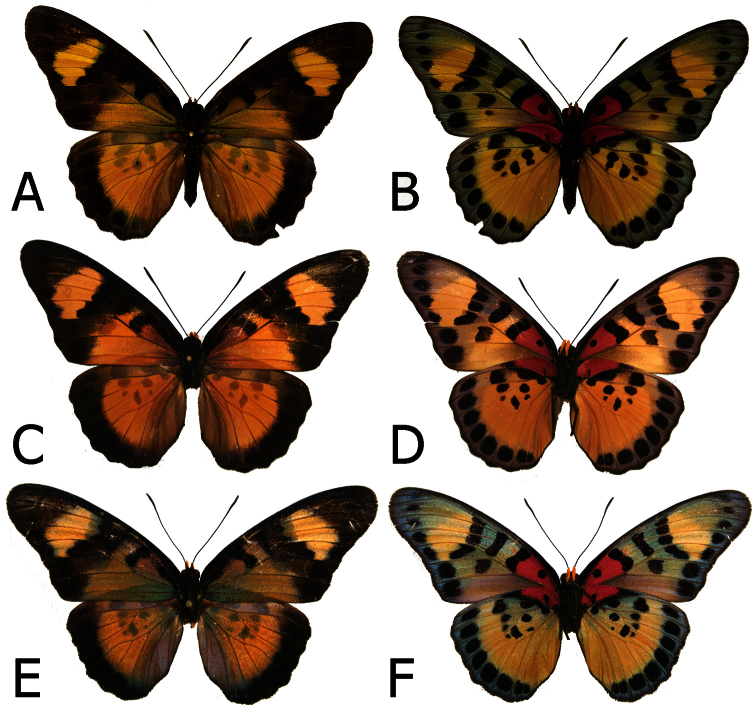
Adults, females: **A** *Euphaedra sarcoptera nipponicorum* Kigoma, Tanzania (dorsum) **B** *Euphaedra sarcoptera nipponicorum* Kigoma, Tanzania (venter) **C** *Euphaedra sarcoptera nipponicorum* Mbandaka, Congo (dorsum) **D** *Euphaedra sarcoptera nipponicorum* Mbandaka, Congo (venter) **E** *Euphaedra sarcoptera nipponicorum* Mbandaka, Congo (dorsum) **F** *Euphaedra sarcoptera nipponicorum* Mbandaka, Congo (venter).

Figs 6C, 6D , 8A–F , 11B, 11D, 11F , 12A, 12C

Najas sarcoptera nipponicorum Carcasson, 1965: 132, fig. 4.
Euphaedra sarcoptera nipponicorum (Carcasson); Ackery et al. 1995: 414.

##### Type-locality.

Ititye Camp, Mihuno, 25 miles east of Kigoma, Western Province, Tanzania.

##### Material examined.

3 ♀: Kasye Forest, Kigoma, Tanzania, III.1990, leg. local dealer, ex coll. P. Boyer, MZUJ; 3 ♂: R.C.A., Bangui, C.A.R., VIII.2000, S. Collins leg.; 1 ♂: R.C.A., Bangui, IX.2000, S. Collins leg., 2 ♂ and 1 ♀: R.C.A., Bangui, IV.2004, S. Collins leg.; 1 ♂: R.C.A., Bomoloto, R. Longamp, VII.1985, S. Collins leg.; 2 ♀: R.C.A., Bangui, VIII.2000, S. Collins leg.; 1 ♀: R.C.A., I.2004, S. Collins leg.; 2 ♂: R.D.C. (NW), Lukolela, Congo River, V.2012; 11 ♂ and 10 ♀: Tanzania, ABRI; 1 ♂: Congo, Province de l’Equateur, Mbandaka, 12.II.1998, prep. genit. 01/04.12.2012, J. Lorenc; 1 ♂: same data, prep. genit. 05/04.1.2012, J. Lorenc; 1 ♀: same data but XII.1997, prep. genit. 08/04.12.2012, J. Lorenc; 1 ♂: Congo, Province de l’Equateur, Environ de Mbandaka – Kuluboku, 27.IV.1995, prep. genit. 06/04.1.2012, J. Lorenc; 1 ♀: same data but 15.XI.1995, prep. genit. 07/04.12.2012, J. Lorenc; 1 ♂: Congo, Province de l’Equateur, Lukolela, 26.X.1993, prep. genit. 03/04.12.2012, J. Lorenc; 1 ♂: same data but 27.X.1993, prep. genit. 04/04.12.2012, J. Lorenc; 1 ♂: same data but XI.1993, PhO; 1 ♂: Sandoa, Katanga, R.D.C., Sandoa, 16.I.1991; 1 ♂: same data but 30.X.1920; 1 ♂: Kafakumba, Katanga, R.D.C., VII.1939; F.G. Overlaet leg.; 2 ♂: same data but II.1939; 1 ♂: same data but IX.1933; 1 ♂: Kinda, Katanga, R.D.C., 31.III.1916; 1 ♂: same data but 31.VIII.1916; 1 ♂: Montamba, 24.V.1916; 1 ♂: Kondué, East Kasai, R.D.C., no date, E. Luja leg.; 1 ♂: Tshilolo, Sankuru, East Kasai, 10.III.1951, Dr. Vallard leg.; 1 ♀: Kafakumba, Katanga, R.D.C., II.1939, F. G. Overlaet leg.; 1 ♀: same data but III.1939; 1 ♀: same data but IV.1939; 1 ♀: same data but IX.1933; 1 ♀: Kinda, 27.II.1916, F. G. Overlaet leg., MRAC.

##### Diagnosis.

Upperside ground colour golden yellow, same as in *ferrea*.

##### Redescription.

**Male** (Figs 6C, 6D): Head, thorax and abdomen same as in of the nominate subspecies. Wings: FW (length: 37–43 mm, mean: 40,3 mm, n=18) with an elongated apical part, and gently convex outer margin; most of wing surface black; costa from base to apex dusted with dark blue; a golden yellow area with a light green suffusion from wing base to two-thirds of anal margin, covering lower half of discal cell; black postbasal and mid discal cell black round spots connected to black costal area; a large, elongated golden yellow subapical patch, extending from subcosta costa, progressively widening to space M2-M3, then narrowing and reaching to vein Cu1, with an irregular basal limit marked by an incision along vein M3, and a diffused outer limit, not reaching outer margin; fringes grey and black. HWD subtriangular with a gently scalloped outer margin; golden yellow, from Rs to costal margin dull brown; submarginal and marginal area black with a strong blue sheen, gradually narrowing from costa, where some 4 mm wide, to tornus, where greenish-blue takes over black and leaves one black spot in Cu1-Cu2 free; fringes grey and black. FWV green yellow, except for diffuse are in mid cell Cu2-1A-2A where free of green and in subapical area where forming a large patch shaped as on the upperside; basal to median area purple; a series of black patches, a rounded postbasal spot, a wide mid cell bar, a similar bar along discal cell distal margin, a series of four, moon-shaped patches along basal limit of a diffused subapical light yellow area, a large moon-shaped patch in mid cell Cu1-Cu2 coupled with a minute spot in Cu2-1A-2A, and a series of submarginal patches, five small from costa to cell M3-Cu1 and two large, roughly oval, in Cu1-Cu2 and Cu2-1A-2A. HWV golden yellow, with an elongated basal purple patch extending from costal margin to Rs to one-fourth costa, edged with black; six to seven black median spots, three or four of which in discal cell, and a row of eight large, roughly oval black submarginal patches; marginal area darker bottle green and black. *Male genitalia*: (Figs 11B, 11D, 11F) Tegumen same length as uncus, with a dome-like dorsal surface; uncus stout, more so than in other subspecies, slightly arched, with a sharp tip pointing downwards; gnathos long, same length as uncus; pedunculus prominent; saccus flattened in lateral view; valvae with a smooth dorsal surface with a blunt distal extremity ending with a small tip; aedeagus as long as valvae tubular and straight, with a sharp distal extremity, longer than in the nominate subspecies or *styx*, approaching the length of *nimbta*,and prominent cornuti.

**Female** (Figs 8A–F): Sexual dimorphism slight except for the larger size of the female (FW length: 40–51 mm, mean: 47,5 mm, n=10). *Female genitalia* (Figs 12A, 12C): Bursa copulatrix very large, larger than the rest of genital body, oval, no signa; ductus bursae wide, of same width throughout, one-third the length of bursa; colliculum short and weakly sclerotized; ductus seminalis joins the colliculum at the entrance of ductus bursae; lamella postvaginalis very small, slat-like; papillae anales three times as long as wide in lateral view, compressed in ventral view, apophyse posteriores one and half as long as the width of papillae anales; von Siebold organ prominent.

**Figure 9.**
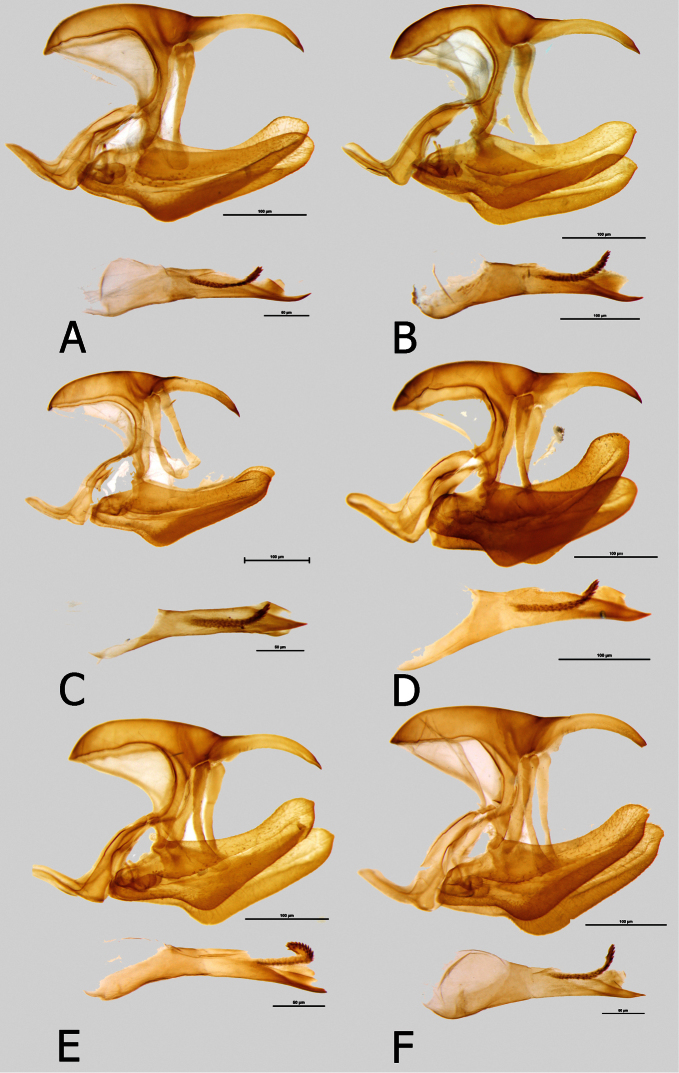
Male genitalia (in lateral view, aedeagus extracted): **A** *Euphaedra cyparissa cyparissa* Guma, Sierra Leone **B** *Euphaedra cyparissa tai* Tai, Ivory Coast **C** *Euphaedra cyparissa nimbina* Mont Nimba, Guinea **D** *Euphaedra cyparissa aurantina* Abengourou, Ivory Coast **E** *Euphaedra cyparissa aurata* Ebogo, Cameroon **F** *Euphaedra cyparissa nominalina* Bangui, Central African Republic.

**Figure 10.**
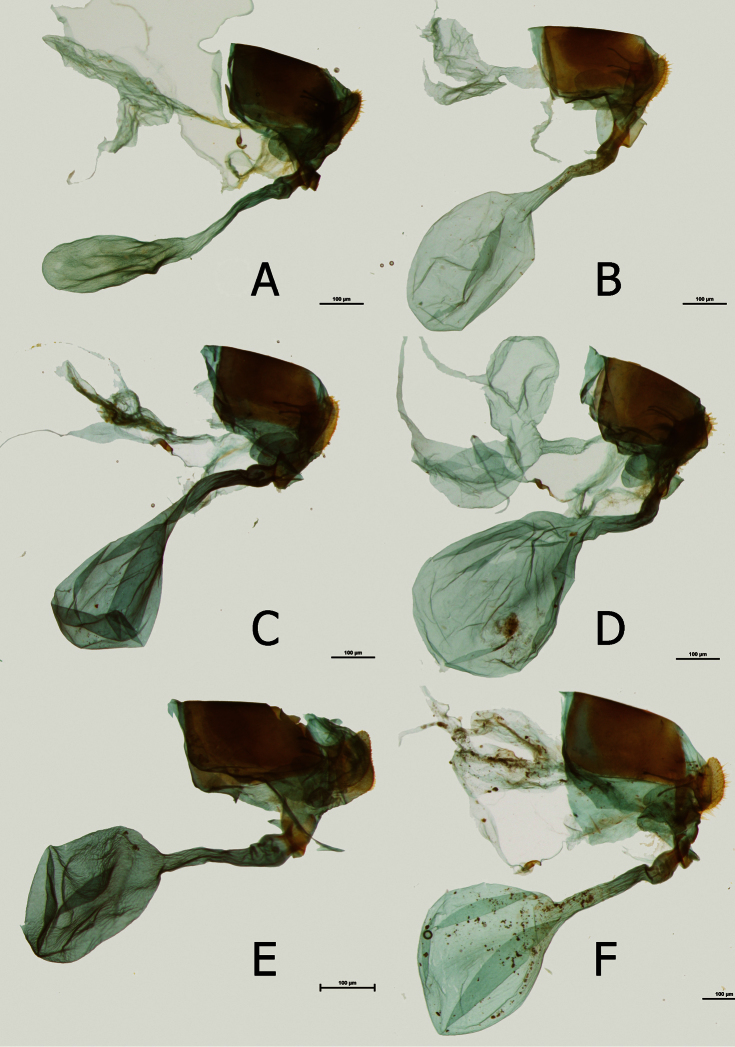
Female genitalia (in lateral view): **A** *Euphaedra cyparissa aurantina* Mpasso, Ghana **E** *cyparissa aurantina* Abengourou, Ivory Coast; 1 ♂: S. Nigeria, Ewohimi, 29.XI.55, J. Boorman; 1 ♂: no label; 2 ♂: Cameroon, Ebogo, Mbalmayo, X.2001; 1 ♂: Cameroon **C** *Euphaedra cyparissa nimbina* Mont Nimba, Guinea **D** *Euphaedra cyparissa tai* Tai, Ivory Coast **E** *Euphaedra cyparissa aurata* Obudu, Nigeria **F** *Euphaedra cyparissa nominalina* Mongorimba, R.C.A.

**Figure 11.**
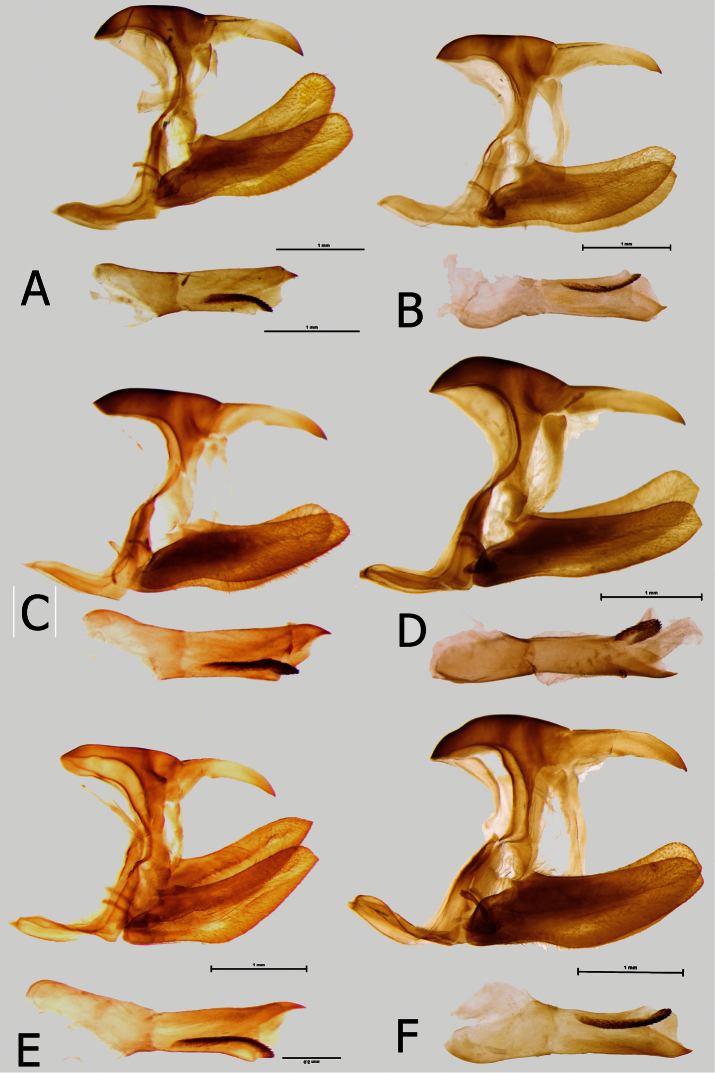
Male genitalia (in lateral view, aedeagus extracted): **A** *Euphaedra sarcoptera sarcoptera* Bibiani, Ghana **B** *Euphaedra sarcoptera nipponicorum* Mbandaka, Congo **C** *Euphaedra sarcoptera styx* Tai, Ivory Coast **D** *Euphaedra sarcoptera nipponicorum* Mbandaka, Congo **E** *Euphaedra sarcoptera ferrea* Mont Nimba, Guinea **F** *Euphaedra sarcoptera nipponicorum* Mbandaka, Congo.

**Figure 12.**
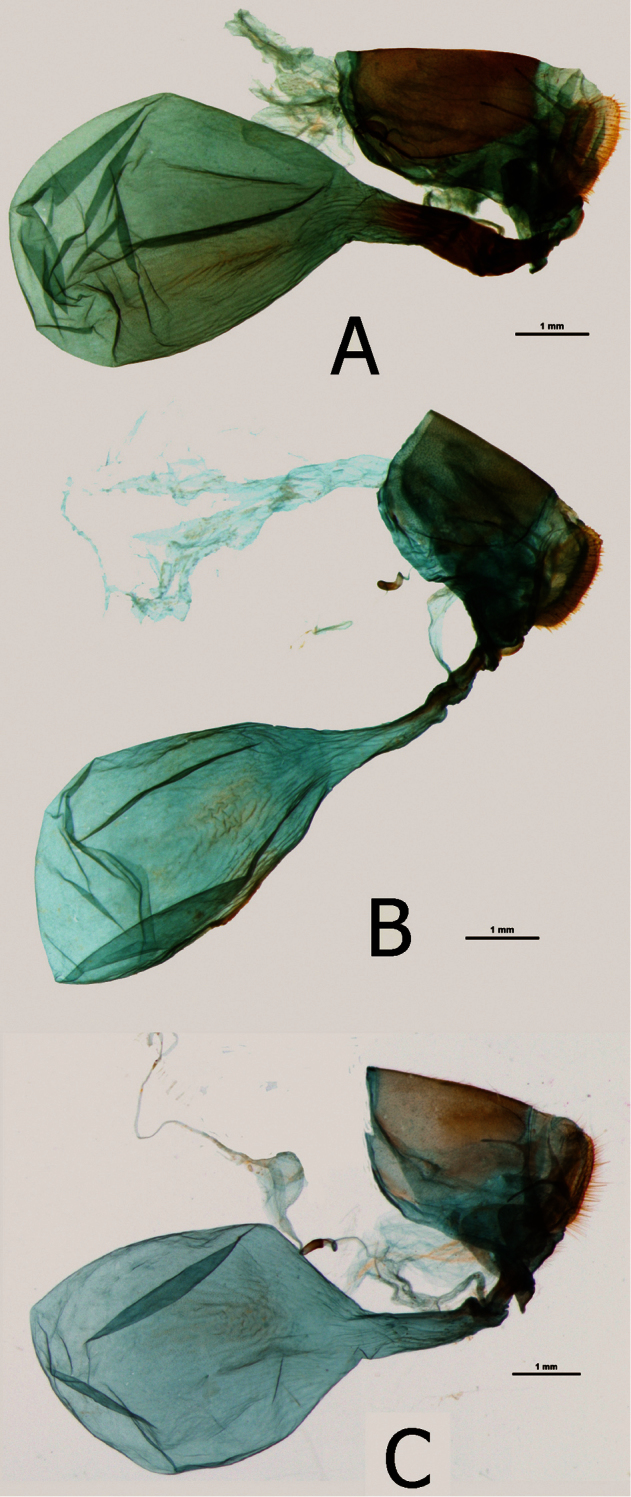
Female genitalia (in lateral view): **A** *Euphaedra sarcoptera nipponicorum* Kigoma, Tanzania **B** *Euphaedra sarcoptera ferrea* Mont Nimba, Guinea **C** *Euphaedra sarcoptera nipponicorum* Mbandaka, Congo.

**Figure 13.**
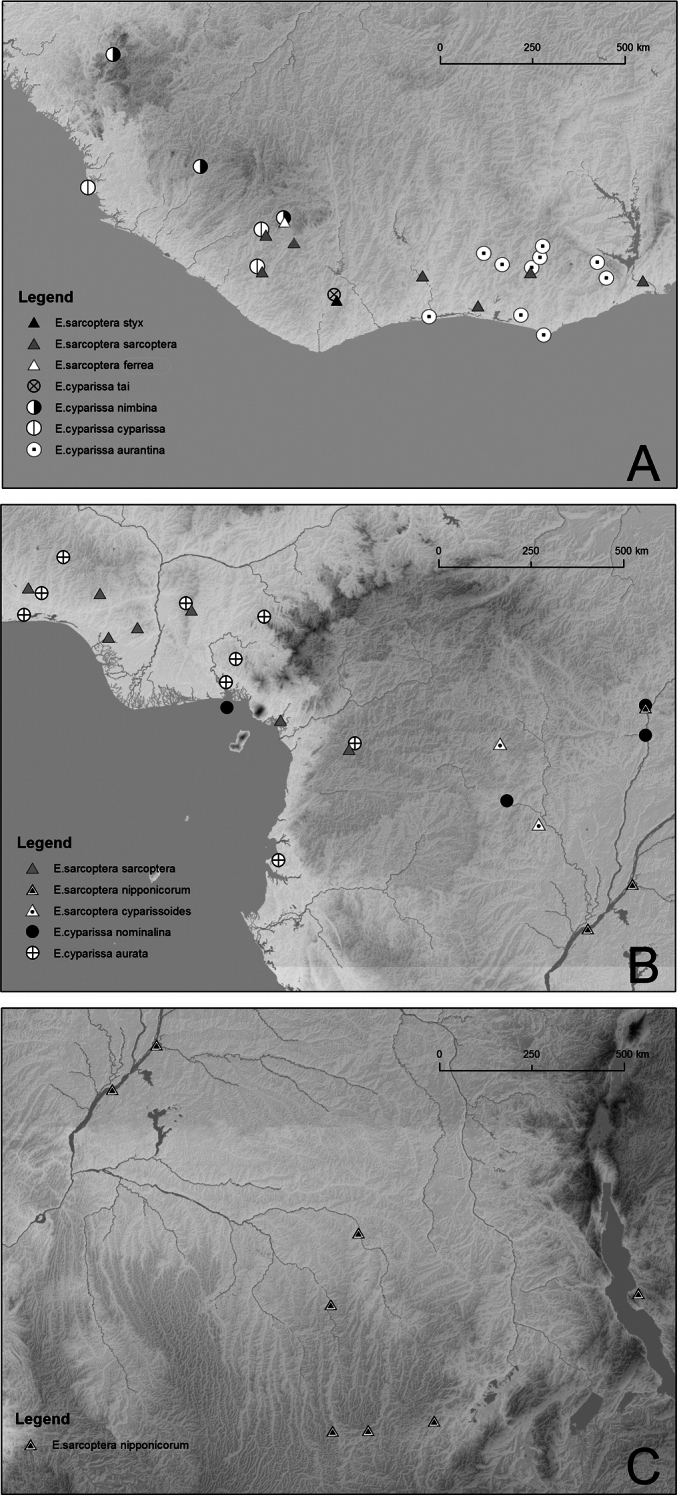
Distribution map: **A** West Africa, west of the Dahomey Gap **B** West Africa east of the Dahomey Gap, and central Africa **C** Congo Basin

##### Range.

This subspecies was described from the now largely destroyed Kasye forest in western Tanzania. It is however widely distributed across the Congo Basin south to Katanga (Shaba). The individuals coming from the area of Mbandaka in the western part of the Congo Basin are attributable to this subspecies. They present however an unusual, apparently individual variation in the ground colour of the upperside, ranging from orange-yellow, through typically golden yellow to yellow with a strong green overcast. Such an apparent individual variation, quite common in some species of *Euphaedra sensu lato*, has been also observed in the Mount Swa population of *Euphaedra sarcoptera*. We refrain from further actions until more thorough sampling in the western Congo Basin confirms the differently patterned individuals are either syntopic or represent parapatric subspecies.

## Discussion

### Distribution and ecology

In terms of geographical range, the two species are largely sympatric, though, as noted above, *Euphaedra sarcoptera* stretches further south and east, into the Congo basin and the eastern shores of Lake Tanganyika. The six subspecies of *Euphaedra cyparissa* and the four of *Euphaedra sarcoptera* are discrete, in the sense of no overlap, and morphologically clearly distinct. Examination of the genitalia makes it clear that only two species are involved.

The apparent gaps in the range of *Euphaedra cyparissa* are puzzling. This is particularly striking in Nigeria where there are (or there were, since most of the western forests have now been logged down) strong populations in Yorubaland. There are however no records from the localities situated further south and east on the western bank of the river Niger. This is despite long-term and intensive collecting in the Okomu forest (Edo) by Wojtusiak, Knoop, Larsen and other researchers, or in Ajebandele (Ondo) by Pyrcz. East of the river Niger the pattern is equally unusual. Only one specimen was collected by Wojtusiak in Nsukka during five years of sampling. Larsen, however, collected several in the Oban Hills. Specimens are known from the Obudu range, and the riverine forests near Calabar. In Ghana and Ivory Coast the species seems to be widespread but local, distributed from Danané in north-western Ivory Coast to the Atewa Range in the Eastern Region of Ghana (Larsen 2006a; Larsen et al. 2007). Larsen (2005, 2006b) reports *Euphaedra cyparissa* from the Volta region but only as older records, probably from the collection of Father Theodor Maessen and we have not seen any specimens collected recently which would confirm its presence in the mountains on the border of Ghana and Togo. It is uncommon in eastern Sierra Leone and it has only recently been found in Liberia (Mount Swa), where it seems to be strongly localized. In its eastern range, *Euphaedra cyparissa* appears to be extremely scarce and local. In Cameroon, records are limited to two specimens from Ebogo, an intensively sampled locality, in the superb ABRI collection, while Vande Weghe (2010) reports it as very rare from a unique locality in northern Gabon. There are more frequent reports from CAR, however, compared to other *Euphaedra sensu lato* this species is far less frequently collected.

*Euphaedra sarcoptera* has a much wider geographic range but its distribution is also apparently disjunct. It is local and rare in Liberia (Mount Swa), while slightly commoner in some localities west of the Dahomey Gap, in particular in Ivory Coast and Ghana. However east of it, in southern Nigeria, with the exception of Sapoba, it is exceedingly rare, more so than its congener *Euphaedra cyparissa*. In fact, in four years of sampling by Wojtusiak in the forests of SE Nigeria only one specimen was collected, whereas the senior author (TP) despite of many years of collecting in the SW never came across it. Lees (1989) mentions it from Korup and most specimens in the BMNH are from the Niger Delta. Also in Cameroon *Euphaedra sarcoptera* is exceedingly seldom encountered. However, in some areas of the western Congo Basin and in Katanga it is locally rather frequently met. There are very few reports from the central Congo basin despite rather extensive collections of other species of *Euphaedra*.

In terms of habitat, the ecological preferences of *Euphaedra cyparissa* are inconsistent. Vande Weghe (2010) states briefly that *Euphaedra cyparissa* is a “deep forest” species, whereas Larsen (2005) considers it a rain forest edge, disturbed forest and even a gallery savannah species. He reports it “in small fragments of forests where few forest floor butterflies survive”. It seems that both authors may in fact be correct. In our experience, most *Euphaedra cyparissa* have been collected on the fringes of secondary or primary mangrove and riverine forests in good condition, whether it be humid forest typical of the localities in Sierra Leone, or much of the Ivory Coast, or the drier inland forests of the eastern Ghana and Nigeria. The size of the forest is less important than the condition of what remains. Furthermore, and curiously, some populations of *Euphaedra cyparissa* appear to be lower montane, in particular *Euphaedra cyparissa nimbina* and the local population of *Euphaedra cyparissa aurata* found in the Obudu massif on the Nigeria-Cameroon border. *Euphaedra sarcoptera* occurs in similar habitats but Larsen (2005) points out that it is more strictly tied to forest than *Euphaedra cyparissa* which is possibly the case for West Africa. In the southern part of the Congo Basin however *Euphaedra sarcoptera* occurs apparently in riverine forests in a predominantly savanna land. Both species have recently been observed hill-topping on forested hilltops in Liberia (Mount Swa), a behaviour not reported for any other *Euphaedra*. Males perch rather high on leaves of trees in the lower canopy level or on the edge of forest gaps (6–15 m high) in the afternoon hours (14–15.00), they chase all passing *Euphaedra*, often involved in intraspecific and occasionally in interspecific interactions. The larval food plants of *Euphaedra cyparissa* and *Euphaedra sarcoptera* are unknown therefore their habitat preferences based on their hosts cannot be asserted.

### Colour pattern convergence

It is interesting to observe that where the two species fly sympatrically, as they do through much of their range, the wing colour markings in one species are closely matched by the other. There are two plausible explanations for that. Either, it is a parallel evolution reflecting an adaptation to a particular forest light structure, a common background. In fact, all green populations are found in rain forest (*Euphaedra cyparissa tai*, *Euphaedra sarcoptera styx*, *Euphaedra cyparissa nominalina*, *Euphaedra sarcoptera cyparissoides*), the yellow ones (*Euphaedra sarcoptera nipponicorum*, *Euphaedra cyparissa ferrea*, *Euphaedra sarcoptera nimbina*) typically occur in drier and often more patchy forests. The second explanation would involve a mimicry scenario, implying that one of the species is the model, whereas the other the mimic. *Euphaedra* are not known to be obnoxious and unpalatable to predators, however the chemistry of this genus has not been investigated so far, so some level of toxicity cannot be ruled out. Such geographic pattern of colour convergence recalls classical examples of Müllerian mimicry among sympatric species and subspecies of many Ithomiines and *Heliconius* in the neotropical region. Pyrcz (in prep.) suggests another explanation for mimicry among palatable species, involving colour convergence to the most abundant pattern in a given area involving the process of anti-apostatic selection.

### The validity of the subgenus *Euphaedrasensu* Hecq

Apart from the slender body and elongated wings, we did not find any other outstanding morphological characters in *Euphaedra cyparissa* and *Euphaedra sarcoptera* that would set them apart from other *Euphaedra sensu lato*. Male and female genitalia are typical of *Euphaedra sensu lato* compared to species of the “*ceres*” group for example (Pyrcz et al. 2011) or to the “*eleus*” group (Pyrcz et al., in prep.). The shape and the configuration of the vesica cornuti of *Euphaedra sarcoptera* and *Euphaedra cyparissa* do not present unequivocal, qualitative characters distinguishing the two species from other *Euphaedra sensu lato*. As between the two species, the external similarity of *Euphaedra cyparissa* and *Euphaedra sarcoptera* in terms of wing shape and colour patterns is striking. There are however significant differentiating characters, apart from the presence or absence of the pinkish basal patch on the FWV. In particular, the antennae of *Euphaedra cyparissa* are chestnut – orange on the underside while those of *Euphaedra sarcoptera* are black. In the male genitalia of *Euphaedra cyparissa*,the tegumen extends considerably basally, which is an exclusive character among the examined species of *Euphaedra sensu lato*, while in *Euphaedra sarcoptera* it is moderately elongated in the same way as in other species of *Euphaedra sensu lato*. The uncus of *Euphaedra cyparissa* is particularly slender and long while that of *Euphaedra sarcoptera* is average in length and thickness for species of *Euphaedra sensu lato*. In the female genitalia *Euphaedra cyparissa* and *Euphaedra sarcoptera* differ more from each other than the subgenus *Euphaedra sensu stricto* from *Euphaedra sensu lato*. The most noticeable character of *Euphaedra sarcoptera* is the particularly large corpus bursae and massive, yet short, ductus bursae, compared to the rather average in size homologous structures of *Euphaedra cyparissa*.

The most interesting trait of *Euphaedra cyparissa* and *Euphaedra sarcoptera* is arguably their unique behaviour, particularly striking for any naturalist with African rain forest experience. Whereas other *Euphaedra sensu lato* fly rapidly very close to the ground, *Euphaedra cyparissa* and *Euphaedra sarcoptera* flutter slowly, often several meters above the ground, and never keep to the understory. Larsen (2005) quotes Fermon’s trapping result in Ghana where she collected many individuals in traps placed at 12 – 25 m above the ground in the forest canopy, which is justified by recent observations of hill-topping behaviour and displaying of males of both species on and above the lower canopy level. The senior author of this paper also observed the courtship behaviour of *Euphaedra cyparissa tai* in Tai National Park Forest (August, 1983), during which the two sexes flew up to some 3–5 metres above the rest of the group and engaged in a typical Nymphalid courtship behaviour (Thornhill and Alcock 1983).

## Conclusion

In the light of this study, previously published articles and the ongoing research on the taxonomy of *Euphaedra sensu lato* (Pyrcz et al., in prep.), the morphological differences between the species in the subgenus *Euphaedra sensu* Hecqand other subgenera of *Euphaedra sensu lato* are insufficient alone to warrant it a separate taxonomic status. We found no characters in the external or genital morphology exclusive of *Euphaedra sarcoptera* and *Euphaedra cyparissa* which could be considered as solid qualitative synapomorphies sustaining its possible monophyly. However, there is a case for retaining the subgenus resting on behavioural grounds. The two species present unique features, involving territoriality and mating strategies, and the occurrence in the forest subcanopy. This study emphasizes as well the need for taking into account also other comparative and phylogenetically valuable characters than those so far available in the research on the genus *Euphaedra sensu lato*.

## Supplementary Material

XML Treatment for Euphaedra cyparissa cyparissa

XML Treatment for Euphaedra cyparissa tai

XML Treatment for Euphaedra cyparissa nimbina

XML Treatment for Euphaedra cyparissa aurantina

XML Treatment for Euphaedra cyparissa aurata

XML Treatment for Euphaedra cyparissa nominalina

XML Treatment for Euphaedra sarcoptera sarcoptera

XML Treatment for Euphaedra sarcoptera styx

XML Treatment for Euphaedra sarcoptera ferrea

XML Treatment for Euphaedra sarcoptera cyparissoides

XML Treatment for Euphaedra sarcoptera nipponicorum
